# Maize Crops Face High Stomatal Uptake During High Exposure to Ozone in an Agroecosystem in the United States Corn Belt

**DOI:** 10.1111/gcb.70952

**Published:** 2026-06-05

**Authors:** Anam M. Khan, Reem Hannun, Elizabeth A. Ainsworth, Lun Gao, Carl J. Bernacchi, Kaiyu Guan, Taylor Pederson, Paul C. Stoy

**Affiliations:** ^1^ Department of Forest and Wildlife Ecology University of Wisconsin‐Madison Madison Wisconsin USA; ^2^ Atmospheric Science Branch NASA Ames Research Center Mountain View California USA; ^3^ Department of Crop Sciences and Plant Biology University of Illinois Urbana‐Champaign Chicago Illinois USA; ^4^ Department of Natural Resources and Environmental Sciences, College of Agricultural, Consumers, and Environmental Sciences University of Illinois Urbana‐Champaign Chicago Illinois USA; ^5^ Agroecosystem Sustainability Center, Institute for Sustainability, Energy, and Environment University of Illinois Urbana‐Champaign Chicago Illinois USA; ^6^ Global Change and Photosynthesis Research Unit USDA ARS Chicago Illinois USA; ^7^ University of Illinois Urbana‐Champaign Chicago Illinois USA; ^8^ Department of Biological Systems Engineering University of Wisconsin‐Madison Madison Wisconsin USA

**Keywords:** eddy covariance, maize, ozone, ozone flux, phytotoxic dose, stomatal conductance

## Abstract

Tropospheric ozone (O_3_) is a phytotoxic air pollutant. The stomatal uptake of O_3_ is a substantial sink of O_3_, but it can create oxidative stress within plants, reducing photosynthesis and crop yields. In seasonally dry climates, stomatal regulation causes temporal decoupling between stomatal conductance and atmospheric O_3_ concentrations protecting vegetation from high stomatal uptake of O_3_ during peak ambient concentrations in the afternoon, but less is known about this temporal decoupling over agricultural fields in continental humid climates. Here, we investigate how maize ( *Zea mays* L.) ecophysiology impacts O_3_ dry deposition and diurnal O_3_ exposure‐dose dynamics at an agricultural field in the central Corn Belt of the United States. We measured the field‐scale eddy covariance flux of O_3_ using a UV‐absorption based instrument, the NASA Rapid Ozone Experiment (ROZE). The stomatal component of total O_3_ flux was estimated with an inversion of the Penman–Monteith equation using observed latent heat flux and a model of stomatal conductance using gross primary productivity. We found that maize stomatal conductance remained high as vapor pressure deficit increased during the afternoon. The diurnal synchrony between O_3_ concentrations and stomatal conductance resulted in high stomatal uptake of O_3_ during peak O_3_ concentrations. The monthly mean stomatal flux reached > 70% of the total O_3_ flux to the land surface during high leaf area index. Furthermore, the total deposition velocity of O_3_ was tightly coupled with stomatal conductance. Our findings suggest that maize ecophysiology in our field in the Corn Belt of the United States couples high O_3_ stomatal uptake with high O_3_ exposure. Furthermore, our study demonstrates the first use of NASA ROZE to measure growing season O_3_ flux over an agricultural field, and NASA ROZE will be crucial for expanding O_3_ flux measurements necessary for studying O_3_ dry deposition and field‐scale phytotoxic dose across other fields.

## Graphical abstract

Tropospheric ozone (O_3_) is a phytotoxic air pollutant. The stomatal uptake of O_3_ is a substantial sink of O_3_, but it can create oxidative stress within plants, reducing photosynthesis and crop yields. In seasonally dry climates, stomatal regulation causes temporal decoupling between stomatal conductance and atmospheric O_3_ concentrations protecting vegetation from high stomatal uptake of O_3_ during peak ambient concentrations in the afternoon, but less is known about this temporal decoupling over agricultural fields in continental humid climates. Here, we investigate how maize (*Zea mays* L.) ecophysiology impacts O_3_ dry deposition and diurnal O_3_ exposure‐dose dynamics at an agricultural field in the central Corn Belt of the United States. We measured the field‐scale eddy covariance flux of O_3_ using a UV‐absorption based instrument, the NASA Rapid Ozone Experiment. Maize stomatal conductance remained high as vapor pressure deficit increased during the afternoon. The diurnal synchrony between O_3_ concentrations and stomatal conductance resulted in high stomatal uptake of O_3_ during peak O_3_ concentrations.

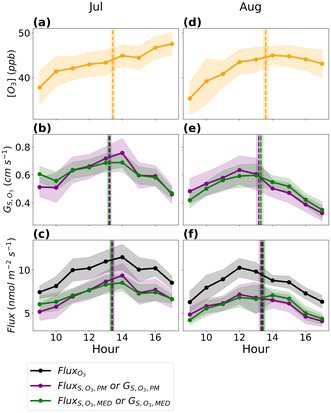

## 1 Introduction

Tropospheric ozone (O_3_) is a phytotoxic air pollutant which is partly removed from the atmosphere through dry deposition. Plants serve as a substantial sink of O_3_ through both stomatal uptake and the chemical destruction of O_3_ on plant cuticles (Wedow et al. 2021). While stomatal uptake removes O_3_ from the atmosphere, it creates oxidative stress in plants causing reductions in photosynthesis, biomass, and crop yields (Reich 1987; Feng et al. 2008, 2022; Ainsworth et al. 2012). When O_3_ diffuses into the intercellular spaces of plants, it degrades to reactive oxygen species (ROS) which can impact primary and secondary carbon metabolism and trigger biochemical pathways involved in antioxidant defense (Kangasjärvi et al. 2005; Heath 2008; Wedow et al. 2021). O_3_ induced bursts of ROS can react with cellular membranes, impact photosynthetic enzymes such as Rubisco, increase mitochondrial respiration, increase the biosynthesis of antioxidant secondary metabolites, and impact stomatal function (Dizengremel 2001; Dizengremel et al. 2009; Wilkinson and Davies 2009; Li et al. 2022; Li, Leakey, et al. 2023). Experimental studies have revealed O_3_ induced losses of 20% in photosynthetic rates for soybean and wheat (Morgan et al. 2003; Feng et al. 2008). Experimental and modeling studies have also demonstrated O_3_ induced reductions in crop yields for rice, wheat, soybean, and maize; however, modeling O_3_ induced yield losses can involve multiple sources of uncertainty, some of which can arise from assumptions about O_3_ dose–response relationships (Ainsworth 2017).

Dose–response relationships are developed in controlled experiments where plants are exposed to heightened levels of O_3_ compared to ambient or charcoal‐filtered air (Uddling et al. 2004; Emberson et al. 2009; Lee et al. 2022). Since the plants are grown in conditions without water stress, which might limit stomatal conductance, the enhanced O_3_ exposure can be assumed to be a reliable indicator of stomatal uptake and hence the dose of O_3_. However, applying dose‐ or exposure‐response relationships to natural conditions can be challenging. On the one hand, crop response to a given dose or exposure is hard to generalize because of the heterogeneity that exists in dose‐ or exposure‐ relationships within and across species and because of the differences in microclimate created by open‐top chamber experiments (Emberson et al. 2009; Ainsworth 2017; Nussbaum and Fuhrer 2000). On the other hand, the dose of O_3_ through the stomata is hard to quantify across crop fields in natural conditions due to the lack of observations that can be used to estimate the stomatal flux of O_3_. As O_3_ concentration data are more widely available, they are used in calculating exposure‐based metrics that have shown dose–response type relationships with yield losses in controlled conditions (Lefohn et al. 2018). Owing to this wider availability of O_3_ concentration measurements, county‐level statistical models predict yield losses in response to exposure‐based metrics, although the stomatal flux of O_3_ more closely reflects the phytotoxic dose that is delivered to the intercellular spaces of crops (McGrath et al. 2015; Hong et al. 2020).

Unlike the controlled conditions under which concentration‐based dose–response relationships were developed, factors such as water stress can limit the stomatal uptake under natural conditions. If ambient O_3_ concentrations remain high under water‐limited stomatal conductance, stomatal uptake becomes decoupled from O_3_ exposure (Panek and Goldstein 2001; Cieslik 2009; Anav et al. 2018). The mismatch between stomatal O_3_ flux and ambient O_3_ concentrations in natural conditions has been observed across forests, grasslands, and croplands (Panek and Goldstein 2001; Cieslik 2004; Stella, Kortner, et al. 2013). In many ecosystems, stomatal conductance and thereby stomatal O_3_ uptake can be limited in the afternoon due to high near‐surface vapor pressure deficit (VPD) when O_3_ concentrations are at their peak (Coyle et al. 2009; Fares, McKay, et al. 2010; Fares et al. 2013; Visser et al. 2021). Seasonally or interannually, times of drought (or seasonally dry times such as in Mediterranean climates) are marked by soil water deficits along with high VPD which can limit stomatal conductance, but again favor the enhancement of O_3_ concentrations due to decreased dry deposition and increased photohemical production (Fares, Goldstein, and Loreto 2010; Emberson et al. 2013; Anav et al. 2018).

Stemming in part from extensive studies of seasonally water‐stressed Mediterranean ecosystems, it is often suggested that reductions in stomatal conductance in response to high VPD or low soil moisture cause temporal decoupling between O_3_ exposure and O_3_ stomatal flux, protecting vegetation from a high dose during peak exposure (Heath et al. 2009; Fares, McKay, et al. 2010). Conversely, stomatal O_3_ flux in a well‐watered rice paddy field and a winter wheat field in China peaked in the afternoon in synchrony with high afternoon O_3_ concentrations (Oue et al. 2008; Wang et al. 2015). However, less is known about the stomatal regulation driven temporal decoupling between O_3_ stomatal dose and O_3_ exposure in natural conditions for maize ( *Zea mays* L.) crops with C_4_ photosynthesis in the United States' maize growing region characterized by warm, humid summers. Here, we present eddy covariance measurements of O_3_ flux over a maize agricultural field near Bondville, IL and analyze the stomatal component of the O_3_ flux to this field. We ask:

1. How does maize ecophysiology impact the stomatal dose of O_3_ through the peak and end of the growing season over an agricultural field in the continental humid, midwestern region of the United States?
2. How does the phytotoxic stomatal dose of O_3_ compare with exposure‐based metrics calculated from O_3_ concentrations at this field?

## 2 Materials and Methods

### 2.1 Data Collection and Processing

All data was collected at an Ameriflux eddy covariance tower at a maize field in central Illinois near Champaign, IL, USA (Meyers 2016). The eddy covariance tower is located at 40°0′22.2186″, −88°17′25.5408″. The field is managed as a rainfed maize/soybean rotation. O_3_ concentrations were measured using the NASA Rapid Ozone Experiment (ROZE) (Hannun et al. 2020). ROZE utilizes UV broadband, cavity enhanced, absorption spectroscopy to achieve high sensitivity to O_3_ thus ensuring high precision. The high flow rates and small footprint of the optical cavity enable rapid flush rates of the sample cell and ensure the high sampling frequency needed for the eddy covariance method (Hannun et al. 2020). ROZE has been validated against chemiluminescence‐based O_3_ concentrations from the NOAA Nitrogen Oxides and Ozone instrument and showed good agreement within the stated uncertainty for each instrument (~6% for ROZE) (Hannun et al. 2020). We collected O_3_ concentrations from ROZE from July 1 to September 21, 2023. The instrument was regularly calibrated in the field with a 2B Technologies (Broomfield, CO, USA) O_3_ calibration source, model 306, in July, August, and September.

CO_2_ and H_2_O concentrations were collected using an open‐path Licor (Lincoln, NE, USA) infrared gas analyzer, LI‐7500DS, at a 10 Hz frequency. Wind velocity was collected using a 3‐axis Gill (Lymington, Hampshire, UK). Windmaster Pro sonic anemometer at a 10 Hz frequency. Instruments were mounted 3.5 m above the ground. Air temperature and humidity were measured using a Vaisala (Vantaa, Finland) HMP 155 probe. Incoming and outgoing shortwave radiation and longwave radiation were measured with a Kipp & Zonen (Sterling, VA, USA) CNR4 net radiometer. Leaf area index (LAI) was measured periodically throughout the growing season using a Licor LAI‐2200C. LAI measurements were taken at three different locations on a sampling day. Therefore, we removed outliers and calculated the average measurement for the day.

### 2.2 Calculating O_3_, CO_2_, and H_2_O Flux and Partitioning Net Ecosystem Exchange

The O_3_, CO_2_, and H_2_O flux were calculated at half‐hourly intervals. For each half‐hour block of high frequency data, spikes were detected and removed, and a double rotation was performed on the wind velocity vector such that mean vertical wind velocity was 0 for the half‐hour (Wilczak et al. 2001). Only half‐hours with no more than 10% missing data were used to calculate the flux. We removed half‐hour fluxes that were flagged as low quality by the steady state test and test of integral turbulence characteristics (SSITC) (Foken and Wichura 1996). For each half‐hour, we calculated the flux of a gas *s* (Flux_*s*_), the sensible heat flux (H, W m^−2^), the latent heat flux (λE W m^−2^), and the friction velocity (u*, m s^−1^) as:

(1) $${\text{Flux}}_{s}=\bar{w^{\prime }s^{\prime }}$$

(2) $$H=\rho c_{p}\bar{{w^{\prime }T^{\prime }}_{\mathrm{air}}}$$

(3) $$\mathrm{\lambda E}=\lambda M_{H_{2}O}{\text{Flux}}_{H_{2}O}10^{-3}$$

(4) $$u^{*}={\left({\bar{u^{\prime }w^{\prime }}}^{2}+{\bar{v^{\prime }w^{\prime }}}^{2}\right)}^{1/4}$$

where $w^{\prime }$ is the fluctuation of vertical wind velocity from the mean (m s^−1^), and $s^{\prime }$ is the fluctuation of molar density of gas s (μmol m^−3^ for O_3_ and μmol m^−3^ for CO_2_ and H_2_O), from the mean, ρ is the air density (kg m^−3^), $c_{p}$ is the specific heat of air (J kg^−1^ g^−1^), *T*_air_ is the air temperature (°C), λ is the latent heat of vaporization (J kg^−1^), $M_{H_{2}O}$ is the molecular weight of H_2_O (kg mol^−1^), $u^{\prime }$ is the fluctuation of horizontal wind velocity from the mean (m s^−1^), and $v^{\prime }$ is the fluctuation of cross wind velocity from the mean (m s^−1^).

The CO_2_ flux is equal to the net ecosystem exchange of CO_2_ (NEE) when storage is negligible. NEE was partitioned into gross primary productivity (GPP) and ecosystem respiration (*R*_eco_) using the nighttime partitioning method in REddyProc (Reichstein et al. 2005; Wutzler et al. 2018). A time‐varying relationship between NEE and air temperature based on nighttime data was developed. The nighttime NEE is assumed to be dominantly due to *R*_eco_, and the relationship is applied to daytime temperatures to estimate *R*_eco_ during the day. GPP was calculated as the residual flux after removing *R*_eco_ from NEE. We removed nighttime observations when friction velocity, $u^{*}$, was low to avoid times when the net CO_2_ flux can be biased low during the nighttime as determined using the $u^{*}$ filter described in Reichstein et al. (2005). Specifically, we fit a piecewise linear regression to detect a change point in the nighttime relationship between $u^{*}$ and NEE. Observations were limited to the hours of 19:00–3:00 during July, August, and September to detect a different change point for each month using month‐specific regressions. We chose the maximum change point from the change points detected for the 3 months. This resulted in a change point of 0.09 m s^−1^. This threshold resulted in the removal of 9% of the total nighttime NEE values during July–September. The Marginal Distribution Sampling algorithm was used to gap‐fill air temperature, NEE, VPD, and incoming shortwave radiation for the purpose of partitioning NEE into GPP and *R*_eco_ (Wutzler et al. 2018). For the subsequent analysis described in Sections 2.3, 2.6, we used only measured values.

### 2.3 O_3_ Flux and Deposition Velocity

The flux of O_3_ is proportional to the concentrations of O_3_ with a deposition velocity, *V*_d_ (m s^−1^), as the proportionality factor (Foken and Kramm 1995):

(5) $${\text{Flux}}_{O_{3}}=V_{d}\left({\left[O_{3}\right]}_{r}-{\left[O_{3}\right]}_{s}\right)$$

where [O_3_]_*r*_ is the O_3_ concentration at the measurement height, and [O_3_]_*s*_ is the O_3_ concentration at the surface. O_3_ concentration at the surface is assumed to be 0. Considering that O_3_ concentrations were measured at our site, and O_3_ flux was calculated using Equation (1), we simply calculated deposition velocity as:

(6) $$V_{d}=\frac{{\text{Flux}}_{O_{3}}}{{\left[O_{3}\right]}_{r}}$$

The deposition velocity of O_3_ consists of an aerodynamic component, a quasi‐laminar boundary layer component, and a surface component. These resistances are in series such that deposition velocity is specified as:

(7) $$V_{d}={\left(R_{a}+R_{b,O_{3}}+R_{c,O_{3}}\right)}^{-1}$$

where $R_{a}$ is the aerodynamic resistance imposed by turbulence above the canopy (s m^−1^), $R_{b,O_{3}}$ is the resistance imposed by a thin layer on the surface where molecular diffusion of gasses plays a larger role in transport under neutral conditions (s m^−1^), and $R_{c,O_{3}}$ is the bulk surface resistance to $O_{3}$ (s m^−1^). $R_{a}$ can be calculated from measured wind speed, *u*, as (Verma 1989; Knauer, El‐Madany, et al. 2018):

(8) $$R_{a}=\frac{u}{u{*}^{2}}$$

The quasi‐laminar boundary layer resistance, $R_{b,O_{3}}$, can be calculated as (Hicks et al. 1987):

(9) $$R_{b,O_{3}}=\frac{2}{{\mathrm{ku}}^{*}}{\left(\frac{\mathrm{Sc}}{\mathrm{Pr}}\right)}^{2/3}$$

where *k* is the von Karman constant (0.4), Sc is the Schmidt number (the ratio of kinematic viscosity to the molecular diffusivity of O_3_) and Pr is the Prandtl number (the ratio of kinematic viscosity to thermal diffusivity). For $R_{b,O_{3}}$, we used the ratio of thermal diffusivity of air (0.2 cm^2^ s^−1^) to the molecular diffusivity of O_3_ in air (0.14 cm^2^ s^−1^) (Massman 1998). Using a single layer canopy model, the bulk surface resistance to O_3_, $R_{c,O_{3}}$, consists of the stomatal and non‐stomatal components and can be broken down into two parallel resistances as:

(10) $$R_{c,O_{3}}={\left(R_{s,O_{3}}^{-1}+R_{\mathrm{ns},O_{3}}^{-1}\right)}^{-1}={\left(G_{s,O_{3}}+G_{\mathrm{ns},O_{3}}\right)}^{-1}$$

where $R_{s,O_{3}}$ is the stomatal resistance to O_3_ (s m^−1^) and $R_{\mathrm{ns},O_{3}}$ is the non‐stomatal resistance to O_3_ (s m^−1^), and $G_{s,O_{3}}$ and $G_{\mathrm{ns},O_{3}}$ are the respective conductance terms.

### 2.4 Isolating the Stomatal Flux of O_3_

We used λE and GPP from the eddy covariance tower to partition $R_{c,O_{3}}$ into the stomatal and non‐stomatal components using two estimates of stomatal conductance to water vapor. We estimated the surface conductance to water vapor, $G_{c,H_{2}O,\mathrm{PM}}$, from λE through an inversion of the evaporation‐resistance formulation of the Penman‐Monteith equation which uses the canopy‐to‐air VPD rather than near‐surface VPD (Monteith 1981; Gerosa et al. 2005, 2007):

(11) $$G_{c,H_{2}O,\mathrm{PM}}^{-1}=R_{c,H_{2}O,\mathrm{PM}}=\frac{\rho c_{p}\left[{\mathrm{VPD}}_{\mathrm{can}}\right]}{\mathrm{\gamma \lambda E}}-\left(R_{a}-R_{b,H_{2}O}\right)$$

where γ is the psychrometric constant (kPa K^−1^), VPD_can_ is the difference in vapor pressure between the surface of the canopy and the measurement height (kPa), $c_{p}$ is the specific heat of air (J kg^−1^ K^−1^), and $R_{b,H_{2}O}$ is the boundary layer resistance to H_2_O (s m^−1^). We assumed saturated conditions at the surface and calculated VPD_can_ as:

(12) $${\mathrm{VPD}}_{\mathrm{can}}=e_{s}\left(T_{s}\right)-e\left(r\right)$$

where $e_{s}\left(T_{s}\right)$ is the saturation vapor pressure at the surface temperature (kPa) and $e\left(r\right)$ is the vapor pressure at the measurement height (kPa). We calculated surface temperature, *T*_s_, as:

(13) $$T_{s}=T_{\mathrm{air}}+\frac{H}{\rho c_{p}}\left(R_{a}+R_{b,H}\right)$$

where $R_{b,H}$ is the boundary layer resistance to heat (s m^−1^). Since the PM inversion calculates the total surface conductance to water vapor, we limit our data to observations when λE is made up largely of transpiration and hence the surface conductance serves as an estimate of stomatal conductance. To do so, observations of λE are limited to times that meet the following criteria. We removed observations during a rain event and 36 h after the rain event. Only daytime observations when solar zenith angle was < 85° were used. We also limited observations to times when relative humidity (RH) was < 80% to avoid non‐stomatal contributions to λE. Through this data filtering, we can take $G_{c,H_{2}O,\mathrm{PM}}$ as an estimate of stomatal conductance. Therefore, $G_{c,H_{2}O,\mathrm{PM}}$ will be referred to as $G_{s,H_{2}O,\mathrm{PM}}$. To further check the validity of this assumption, we partitioned λE into transpiration and non‐stomatal evaporation components using the conditional eddy covariance algorithm of Zahn et al. (2022).

To calculate a second stomatal conductance estimate based on GPP, we used a leaf‐level stomatal conductance model (Medlyn et al. 2011) which was recently applied to the eddy covariance scale (Medlyn et al. 2017; Knauer, Zaehle, et al. 2018):

(14) $$G_{s,H_{2}O,\mathrm{MED}}=G_{o}+1.6\;\left(1+\frac{G_{1}}{\sqrt{{\mathrm{VPD}}_{\mathrm{can}}}}\right)\frac{\mathrm{GPP}}{C_{a}}$$

where $C_{a}$ is the ambient ${\mathrm{CO}}_{2}$ concentration. We used λE‐based estimates of stomatal conductance from the PM inversion, $G_{s,H_{2}O,\mathrm{PM}}$, to fit the *G*_0_ and *G*_1_ parameters using nonlinear least‐squares. O_3_ uptake can directly impact stomatal conductance. This should not pose a problem for $G_{s,H_{2}O,\mathrm{PM}}$ because it is an inversion using observed λE. Any environmental condition that is impacting stomatal conductance will be captured in the observed λE. However, many conditions, such as stomatal uptake of O_3_, might cause a varying relationship between photosynthesis and stomatal conductance (Lombardozzi et al. 2012). In this case, the Medlyn et al. (2011) parameters are likely to vary as the slope of the relationship changes. Therefore, we fit a separate *G*_0_ and *G*_1_ parameter every 10 days when at least 20 observations were available. We used 20–127 half‐hour observations to fit the *G*_0_ and *G*_1_ every 10 days.

The stomatal conductance to O_3_, $G_{s,O_{3}}$, was calculated from both the GPP‐based $G_{s,H_{2}O,\mathrm{MED}}$, and the λE‐based $G_{s,H_{2}O,\mathrm{PM}}$. We calculated $G_{s,O_{3}}$ from the two estimates using the ratio of O_3_ diffusivity to H_2_O diffusivity, set as 0.61, as:

(15) $$G_{s,H_{2}O,\mathrm{MED}}=0.61G_{s,H_{2}O,\mathrm{MED}}$$

(16) $$G_{s,H_{2}O,\mathrm{PM}}=0.61G_{s,H_{2}O,\mathrm{PM}}$$

Finally, the two estimates of the stomatal flux of O_3_ were calculated as:

(17) $${\text{Flux}}_{s,O_{3},\mathrm{MED}}=\frac{G_{s,O_{3},\mathrm{MED}}}{G_{c,O_{3}}}V_{d}{\left[O_{3}\right]}_{r}$$

(18) $${\text{Flux}}_{s,O_{3},\mathrm{PM}}=\frac{G_{s,O_{3},\mathrm{PM}}}{G_{c,O_{3}}}V_{d}{\left[O_{3}\right]}_{r}$$

We limited observations to times when $\frac{G_{s,O_{3}}}{G_{c,O_{3}}}$ was between 0 and 1 because these times represent when the stomatal conductance to O_3_ is constrained within the bulk surface conductance to O_3_. The surface conductance to O_3_ is derived from independent measurements of a third gas, O_3_, sharing a stomatal pathway in addition to CO_2_ and H_2_O. Equations (17) and (18) also ensure that we used the O_3_ concentrations at the top of the canopy for calculating the stomatal flux (Gerosa et al. 2005). Lastly, we removed ${\text{Flux}}_{O_{3}}$ observations that were unusually low and co‐occurred with observations of ${\text{Flux}}_{{\mathrm{CO}}_{2}}$ and ${\text{Flux}}_{H_{2}O}$ that were flagged by the SSITC. The data available after all filters described in Sections 2.2, 2.4 is displayed in Figures S2–S4. A diagram summarizing the methods from Equations ((11), (12), (13), (14), (15), (16), (17), (18)) is provided in Figure S1.

To analyze the distributions of $G_{s,O_{3}}$ across the values of ${\mathrm{VPD}}_{\text{canopy}‐\mathrm{air}}$ and atmospheric stability that occurred during July and August, we calculated atmospheric stability as:

(19) $$\frac{r-d}{L}$$

where *r* is the measurement height, *d* is the zero plane displacement, and *L* is the Monin‐Obukhov length.

### 2.5 Diurnal Centroids

To study diurnal synchrony among O_3_ concentrations, the total flux of O_3_, the two estimates of stomatal conductance, $G_{s,O_{3},\mathrm{MED}}$ and $G_{s,O_{3},\mathrm{PM}}$, and the two estimates of stomatal flux of O_3_, ${\text{Flux}}_{s,O_{3},\mathrm{MED}}$ and ${\text{Flux}}_{\sigma ,O_{3},\mathrm{PM}}$, we calculated the daily diurnal centroid for each of these variables and compared the diurnal centroids among the variables. Diurnal centroids have been used to study the diurnal (mis)alignment of carbon and water flux across various ecosystems (Wilson et al. 2003; Nelson et al. 2018; Khan et al. 2022; Li, Ryu, et al. 2023), and they represent the variable weighted hour of the day. A diurnal centroid for a given variable was calculated as:

(20) $$\text{Diurnal}\;{\text{Centroid}}_{\mathrm{var}}=\frac{\sum_{t=7}^{17}{\mathrm{Var}}_{t}t}{\sum_{t=7}^{17}{\mathrm{Var}}_{t}}$$

where *t* is the time in decimal hours from daylight hours of 7–17 and Var_*t*_ is the value of a given variable at time *t*. We also tested calculating diurnal centroids with a daylight window of 9:00–17:00. Diurnal centroids were calculated using half‐hour data during any day when at least 9 data points were available. A variable's diurnal centroid past the 12th hour of the day indicates a shift of high values of the variable towards the afternoon. For our comparison of diurnal centroids among the variables, we computed the mean and standard error of the mean diurnal centroid for each month for each variable. The alignment of diurnal centroids between multiple variables indicates that the high values of the variables coincide during the day.

### 2.6 O_3_ Exposure and Phytotoxic O_3_ Dose

We calculated two exposure‐based metrics of O_3_ risk assessment and the flux‐based phytotoxic stomatal O_3_ dose over 6 nmol m^−2^ s^−1^ (POD_6_) (Lefohn et al. 2018). The exposure‐based metrics we calculated were the accumulated half‐hourly O_3_ concentration over a threshold of 40 ppb (AOT40) and W126 (Lefohn et al. 2018). Each metric was summed daily for daily accumulated exposure or dose and summed across July and August for an overall accumulated exposure or dose. Daily POD_6_ was calculated as:

(21) $${\mathrm{POD}}_{6}=\sum_{i=1}^{h}\max\left[0,{\text{Flux}}_{s,O_{3},i}-6\right]\;1800$$

where *h* is the number of daytime half‐hours in a day. We calculated two estimates of ${\mathrm{POD}}_{6}$ using ${\text{Flux}}_{s,O_{3},\mathrm{MED}}$ and ${\text{Flux}}_{s,O_{3},\mathrm{PM}}$. Half‐hourly O_3_ concentrations were used to calculate AOT40 and W126. The daily AOT40 was calculated as:

(22) $$\mathrm{AOT}40=\sum_{i=1}^{h}\max\left[0,{\left[O_{3}\right]}_{r,i}-40\right]$$

W126 was calculated as:

(23) $$W126=\sum_{i=1}^{h}W_{i}{\left[O_{3}\right]}_{r,i}$$

where *W*_*i*_ is calculated as:

(24) $$W_{i}=\frac{1}{1+{\mathrm{Me}}^{-A{\left[O_{3}\right]}_{r,i}/1000}}$$

where *M* is 4403 and *A* is 126 (Lefohn et al. 2018). POD_6_, AOT40, and W126 were summed over July and August. This is the case for both the central analysis described above and the sensitivity analysis described below. These months will be referred to as the study period. Evaporation made up a larger portion of the latent heat flux during September, and the estimates of stomatal conductance were less reliable during this time. Since there are missing observations during the study period and the study period does not consist of the entire growing season, the accumulated exposure and dose estimates are conservative estimates. The crops accumulated a larger dosage of and exposure to O_3_ during the entire season if we consider that the accumulation started before measurements started, and the accumulation continued during missing observations. Furthermore, since AOT_40_ and W126 require only O_3_ concentrations, a larger number of days is available to calculate AOT40 and W126. Below, we report accumulated AOT40 and W126 based on the 62 days of data that were available to calculate these metrics, and we report accumulated POD_6_ using 34 days of data available to calculate POD_MED_ and 32 days of data available to calculate POD_PM_. However, we also report the accumulated AOT40 and W126 using the same number of days that were used to calculate POD_MED_ and POD_PM_. The accumulated AOT40 and W126 are inevitably lower when the days to calculate them are limited to the same days as POD_MED_ and POD_PM_, and this estimate of AOT40 and W126 should only be used to assess the impact of a lower sample size. The AOT40 and W126 that are reported using the 62 days is closer to the actual AOT40 and W126 experienced by the field.

We conducted a sensitivity analysis to assess the impacts of data filtering on the calculation of accumulated POD_6_, AOT40, and W126. Since $G_{s,O_{3},\mathrm{MED}}$ and $G_{s,O_{3},\mathrm{PM}}$ are used to calculate POD_6_, the data filtering described in Section 2.4 limits the number of days that can used to calculate the accumulated POD_6_ across July and August. Therefore, we tested the impacts of data filtering from the post precipitation and relative humidity filters described in Section 2.4. We tested 5 post precipitation time windows by removing observations 24, 36, 48, 60, and 72 h after a precipitation event. We tested these post precipitation time windows with 3 relative humidity thresholds (0.7, 0.8, and 0.9) to develop uncertainty bounds for accumulated POD_6_, AOT40, and W126. This resulted in 15 different data filters. This also allowed us to assess how the uncertainty, induced by data filtering, in the parameter estimation of *G*_0_ and *G*_1_ to calculate $G_{s,O_{3},\mathrm{MED}}$ using Equation (14) propagated to the calculation of accumulated POD_6_, AOT40, and W126. To do this, we estimated accumulated POD_6_, AOT40, and W126 using the methods described in Sections 2.4 and 2.6. However, for each of the 15 data filters, we filtered out data according to the filter. This resulted in either a larger or smaller sample size, given a certain data filter compared to the data filter described in Section 2.4. This allowed us to assess the impacts of data filtering on parameter estimation and develop uncertainty bounds for accumulated POD_6_, AOT40, and W126. For each data filter tested, POD_6_, AOT40, and W126 were calculated using the same number of days. Therefore, there are 2 separate uncertainty bounds reported for AOT40, and W126 according to the number of days used to calculate total POD_6_ and POD_MED_. Finally, for each of the 15 data filters, we estimated a moving *G*_0_ and *G*_1_ every 10 days and a season‐wide fixed *G*_0_ and *G*_1_ and propagated these parameter estimations to the final calculation of accumulated POD_6_.

## 3 Results

### 3.1 Site Conditions and the Response of O_3_ Concentrations, O_3_ Flux, NEE, λE, and Stomatal Conductance to Air Temperature and Vapor Pressure Deficit

**FIGURE 1:**
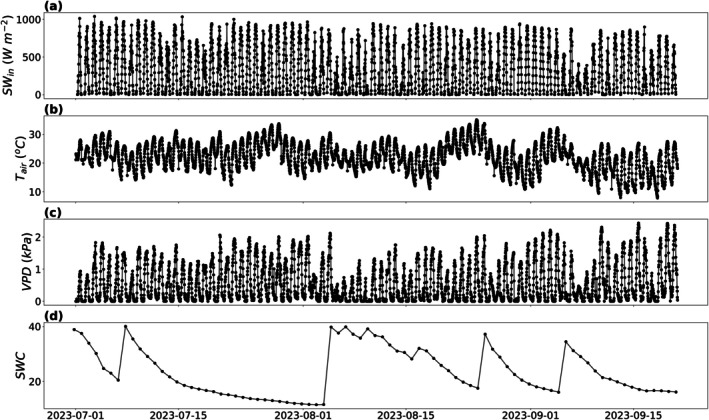
Incoming shortwave radiation (${\mathrm{SW}}_{\text{in}}$; a), air temperature ($T_{\mathrm{air}}$; b), vapor pressure deficit (VPD; c), and volumetric soil water content (SWC; d) at the eddy covariance site in central Illinois near Champaign, IL.

During the data collection, the field experienced many sunny days with high temperatures ideal for both O_3_ production and high C4 maize photosynthetic activity (Figure 1a,b). Figures S2–S4 display the days with valid data and the percent of daily and diurnal data available after the data filters described in Sections 2.2 and 2.4 were applied. The average minimum diel temperature was 17.81°C during July, 17.06°C during August, and 13.50°C during September. The average maximum diel temperature was 28.70°C during July, 27.77°C during August, and 25.78°C during September, with temperatures reaching as high as 33°C and 35°C during certain days in July and August respectively. The mean VPD was 0.68 kPa during July, 0.57 kPa during August, and 0.75 kPa during September (Figure 1c). The field experienced a number of precipitation events during the study period with sharp increases in the soil water content (Figure 1d). LAI remained near stable around 4 m^2^ m^−2^ during July and August with certain surveyed locations reaching an LAI as high as 5.65 m^2^ m^−2^ (Figure S5a). The canopy height followed a similar pattern, reaching a peak height of 2.05 m by mid‐July (Figure S5b).

**FIGURE 2:**
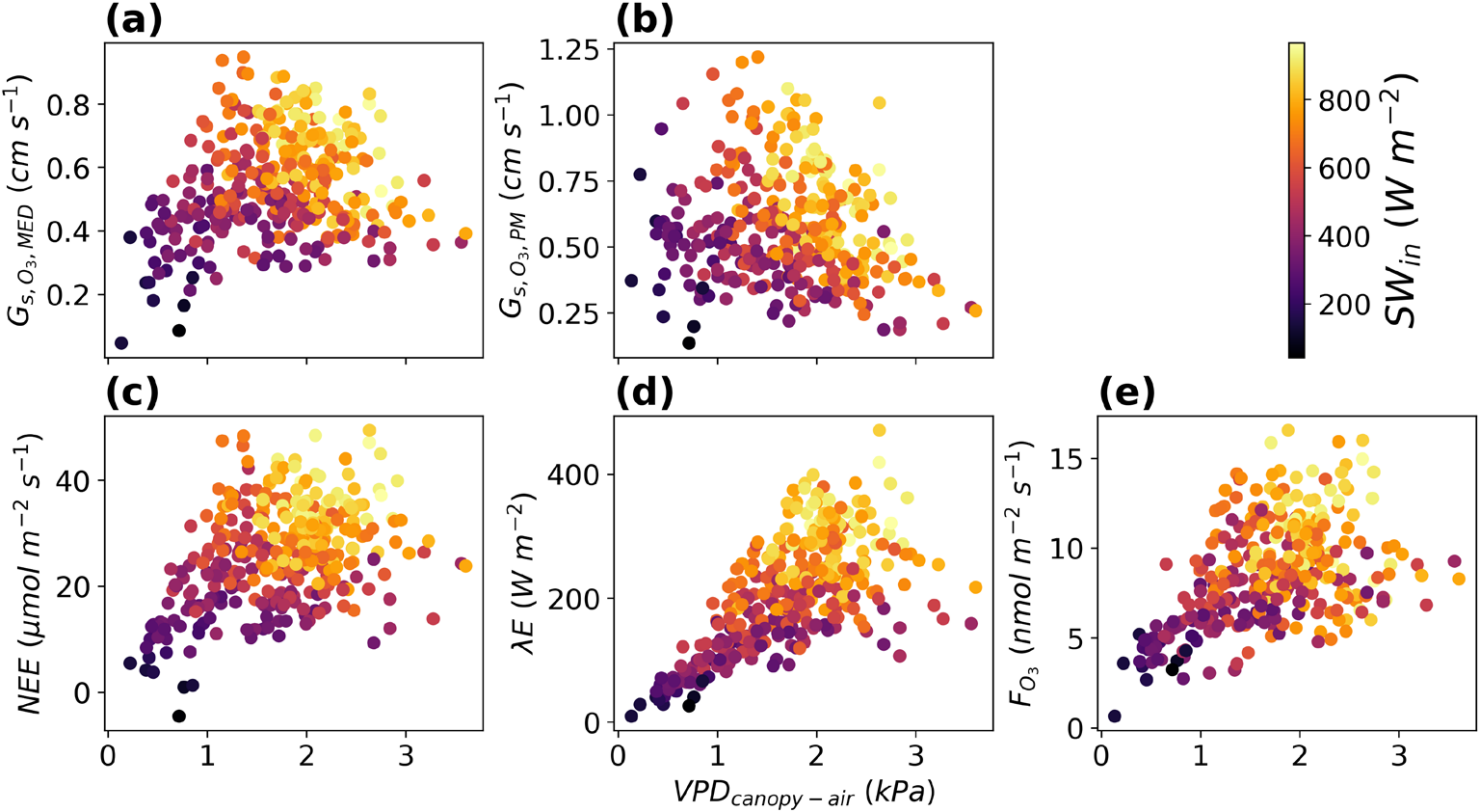
Half‐hour daytime $G_{s,O_{3},\mathrm{MED}}$ (a), $G_{s,O_{3},\mathrm{PM}}$ (b), net ecosystem exchange of CO_2_ (NEE; c), latent heat flux (λE; d), and O_3_ flux ($F_{O_{3}}$; e) plotted against canopy to air vapor pressure deficit (${\mathrm{VPD}}_{\text{canopy}‐\mathrm{air}}$). Dots are colored by values of incoming shortwave radiation (${\mathrm{SW}}_{\text{in}}$). The data displayed does not include data collected during September. NEE is displayed as positive when the land is a net sink of CO_2_.

Transpiration made up over 75% of total evapotranspiration at our field during the daytime in July and August. Therefore, the surface conductance to water vapor from the PM inversion largely consisted of stomatal conductance. We found that high evapotranspiration, CO_2_ assimilation, and O_3_ flux coincided with high shortwave radiation during July and August (Figure 2). NEE is displayed as positive when the crop field served as a net sink of CO_2_. We did not find a decrease in NEE, λE, and O_3_ flux during times of high ${\mathrm{VPD}}_{\text{canopy}‐\mathrm{air}}$ at this field (Figure 2). Stomata remained open at high ${\mathrm{VPD}}_{\text{canopy}‐\mathrm{air}}$ with $G_{s,O_{3}}$ > 0.2 cm s^−1^ during most of July and August from both estimates of $G_{s,O_{3}}$ (Figure 2). The findings are not impacted by atmospheric stability or wind direction (Figures S6 and S7). $G_{s,O_{3}}$ remained above 0.2 cm s^−1^ during the various atmospheric stability values that occurred during the daytime (Figure S6).

During times of high solar radiation (${\mathrm{SW}}_{\text{in}}$ > 600 W m^−2^), $G_{s,O_{3},\mathrm{PM}}$ and $G_{s,O_{3},\mathrm{MED}}$ declined with increasing ${\mathrm{VPD}}_{\text{canopy}‐\mathrm{air}}$, but both estimates of stomatal conductance remained > 0.2 cm s^−1^ during the highest ${\mathrm{VPD}}_{\text{canopy}‐\mathrm{air}}$ experienced during July and August. The highest ${\mathrm{VPD}}_{\text{canopy}‐\mathrm{air}}$ among the data displayed in Figure 2 was 5.34 kPa. There are only two half‐hour data points when ${\mathrm{VPD}}_{\text{canopy}‐\mathrm{air}}$ was > 4 kPa, and these two points were removed for Figure 2. However, the findings from Figure 2 are not impacted by these two data points. There was no reduction in NEE, λE, and total O_3_ flux at these ${\mathrm{VPD}}_{\text{canopy}‐\mathrm{air}}$ values. $G_{s,O_{3},\mathrm{MED}}$ was 0.47 cm s^−1^ when ${\mathrm{VPD}}_{\text{canopy}‐\mathrm{air}}$ was 4 kPa and 0.34 cm s^−1^ when ${\mathrm{VPD}}_{\text{canopy}‐\mathrm{air}}$ was 5.34 kPa. $G_{s,O_{3},\mathrm{PM}}$ was 0.29 cm s^−1^ when ${\mathrm{VPD}}_{\text{canopy}‐\mathrm{air}}$ was 4 kPa and 0.25 cm s^−1^ when ${\mathrm{VPD}}_{\text{canopy}‐\mathrm{air}}$ was 5.34 kPa. At a ${\mathrm{VPD}}_{\text{canopy}‐\mathrm{air}}$ of 4 kPa, the NEE was 35.96 μmol m^−2^ s^−1^, the λE was 261.14 W m^−2^, and 11 nmol m^−2^ s^−1^. At a ${\mathrm{VPD}}_{\text{canopy}‐\mathrm{air}}$ of 5.34 kPa, the NEE was 28.19 μmol m^−2^ s^−1^, the λE was 209.73 W m^−2^, and 9.09 nmol m^−2^ s^−1^. Both $G_{s,O_{3},\mathrm{PM}}$ and $G_{s,O_{3},\mathrm{MED}}$ were above 0.2 cm s^−1^ when ${\mathrm{VPD}}_{\text{canopy}‐\mathrm{air}}$ was 4 and 5.34 kPa. Distributions of $G_{s,O_{3},\mathrm{MED}}$ and $G_{s,O_{3},\mathrm{PM}}$ binned by air temperature and incoming shortwave radiation reveal that for a given temperature range, $G_{s,O_{3},\mathrm{MED}}$ and $G_{s,O_{3},\mathrm{PM}}$ increase with increasing shortwave radiation (Figure S8). Furthermore, distributions of $G_{s,O_{3},\mathrm{MED}}$ and $G_{s,O_{3},\mathrm{PM}}$ binned by ${\mathrm{VPD}}_{\text{canopy}‐\mathrm{air}}$ and incoming shortwave radiation reveal that for a given ${\mathrm{VPD}}_{\text{canopy}‐\mathrm{air}}$ range, $G_{s,O_{3},\mathrm{MED}}$ and $G_{s,O_{3},\mathrm{PM}}$ increase with increasing shortwave radiation (Figure S9). This suggests that within the range of air temperature and ${\mathrm{VPD}}_{\text{canopy}‐\mathrm{air}}$ experienced by the field, incoming shortwave radiation is a prominent limiting factor for *G*_*s*_ at the diurnal scale.

### 3.2 The Stomatal Component of O_3_ Flux

The mean flux of O_3_, the stomatal flux of O_3_, the deposition velocity of O_3_, and the stomatal conductance to O_3_ all declined from July to September. The mean flux of O_3_ was the highest in July (9.76 ± 0.23 nmol m^−2^ s^−1^) and declined through August (8.24 ± 0.19 nmol m^−2^ s^−1^) and into September (5.08 ± 0.12 nmol m^−2^ s^−1^) (Table 1). The mean deposition velocity of O_3_ also peaked in July (0.61 ± 0.01 cm s^−1^) and declined through August (0.53 ± 0.01 cm s^−1^) and into September (0.33 ± 0.01 cm s^−1^) (Table 1). Both estimates of the stomatal flux of O_3_ followed a similar pattern. The highest stomatal flux using ${\text{Flux}}_{s,O_{3},\mathrm{MED}}$ was in July (7.27 ± 0.17 nmol m^−2^ s^−1^) declining through August (5.99 ± 0.17 nmol m^−2^ s^−1^) and into September (3.04 ± 0.11 nmol m^−2^ s^−1^) (Table 1). The highest stomatal flux using ${\text{Flux}}_{s,O_{3},\mathrm{PM}}$ was in July (7.34 ± 0.22 nmol m^−2^ s^−1^) declining through August (5.88 ± 0.18 nmol m^−2^ s^−1^) and into September (2.68 ± 0.12 nmol m^−2^ s^−1^) (Table 1). The stomatal flux of O_3_ made up the highest portion of the total flux on average during July using both ${\text{Flux}}_{s,O_{3},\mathrm{MED}}$ (0.76 ± 0.01) and ${\text{Flux}}_{s,O_{3},\mathrm{PM}}$ (0.75 ± 0.01). We found that the mean stomatal fraction of the total flux of O_3_ was 0.73 ± 0.01 in August and 0.60 ± 0.02 in September using ${\text{Flux}}_{s,O_{3},\mathrm{MED}}$. The mean stomatal fraction of the total flux of O_3_ was 0.72 ± 0.02 in August and 0.53 ± 0.02 in September using ${\text{Flux}}_{s,O_{3},\mathrm{PM}}$ (Table 1).

**TABLE 1:** The mean, standard error of the mean (SEM), and sample size (*n*) for the deposition velocity of O_3_ ($V_{d,O_{3}}$), total flux of O_3_ (${\text{Flux}}_{O_{3}}$), the stomatal flux of O_3_ (${\text{Flux}}_{s,O_{3},\mathrm{MED}}$ and ${\text{Flux}}_{s,O_{3},\mathrm{PM}}$), and the stomatal fraction of total O_3_ flux $\left(\frac{{\text{Flux}}_{s,O_{3},\mathrm{MED}}}{{\text{Flux}}_{O_{3}}}\right)$ and $\left(\frac{{\text{Flux}}_{s,O_{3},\mathrm{PM}}}{{\text{Flux}}_{O_{3}}}\right)$.

Month	$V_{d,O_{3}}$ (cm s^−1^)		${\text{Flux}}_{O_{3}}$ (nmol m^−2^ s^−1^)		${\text{Flux}}_{s,O_{3},\mathrm{MED}}$ (nmol m^−2^ s^−1^)		${\text{Flux}}_{s,O_{3},\mathrm{PM}}$ (nmol m^−2^ s^−1^)		$\frac{{\text{Flux}}_{s,O_{3},\mathrm{MED}}}{{\text{Flux}}_{O_{3}}}$		$\frac{{\text{Flux}}_{s,O_{3},\mathrm{PM}}}{{\text{Flux}}_{O_{3}}}$		*n*
	Mean	SEM	Mean	SEM	Mean	SEM	Mean	SEM	Mean	SEM	Mean	SEM	
Jul	0.61	0.01	9.76	0.23	7.27	0.17	7.34	0.22	0.76	0.01	0.75	0.01	147
Aug	0.53	0.01	8.24	0.19	5.99	0.17	5.88	0.18	0.73	0.01	0.72	0.02	132
Sep	0.33	0.01	5.08	0.12	3.04	0.11	2.68	0.12	0.60	0.02	0.53	0.02	91

**FIGURE 3:**
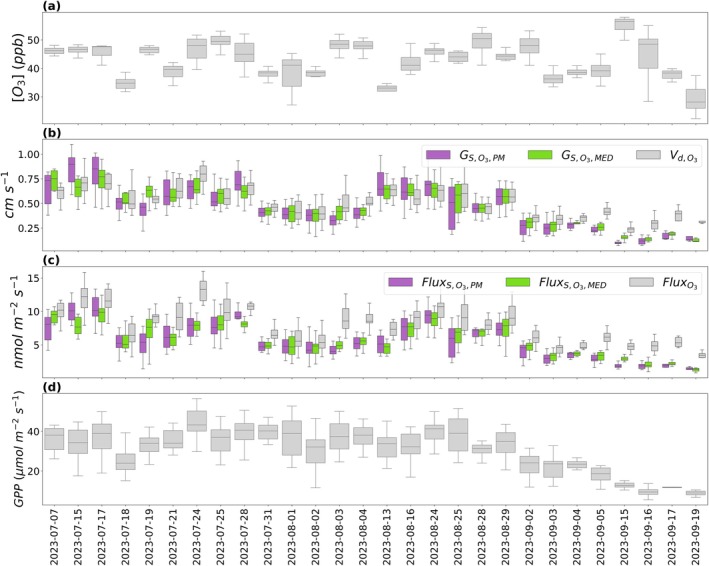
O_3_ concentrations (a), deposition velocity of O_3_ ($V_{d,O_{3}}$; b), stomatal conductance to O_3_ (${\text{Flux}}_{s,O_{3},\mathrm{MED}}$ and ${\text{Flux}}_{s,O_{3},\mathrm{PM}}$; b), total flux of O_3_ (${\text{Flux}}_{O_{3}}$; c), stomatal flux of O_3_ (${\text{Flux}}_{s,O_{3},\mathrm{MED}}$ and ${\text{Flux}}_{s,O_{3},\mathrm{PM}}$; c), and gross primary productivity (GPP; d) by day. Only days with ≥ 5 samples are displayed. The boxes display the interquartile range (IQR) with the median marked with a horizontal line inside the box. The whiskers extend 1.5 IQR on either side of the box. Outliers were removed.

Daily daytime median O_3_ concentration ranged from 30 to 50 ppb with a few days with median concentrations outside of this range (Figure 3a). There was significant day‐to‐day variability in O_3_ concentrations within the 30–50 ppb range. The deposition velocity of O_3_ followed the day‐to‐day course of stomatal conductance through the study period (Figure 3b). The day‐to‐day variability of the total and the stomatal flux of O_3_ followed the variability of GPP and stomatal conductance (Figure 3b–d). An exception to this occurred during September when stomatal conductance and GPP began to decline with corresponding declines in stomatal O_3_ flux, but the O_3_ deposition velocity and flux did not decline as sharply. The non‐stomatal flux made up a larger portion of total O_3_ flux during September compared to July and August due to declining green leaf area, total field‐scale stomatal conductance, and GPP (Figure S5a and Figure 3c,d).

We focus primarily on data from July and August for the remaining results and discussion because our observations during September are limited. However, results from September can be found in the Supporting Information, and we briefly discuss them below. The canopy began entering into senescence in September, and limiting our remaining discussion to July and August ensures that we discuss the role of maize ecophysiology during peak green LAI and growing season. Furthermore, transpiration made up a declining proportion of evapotranspiration during September, making stomatal conductance estimates less reliable.

**FIGURE 4:**
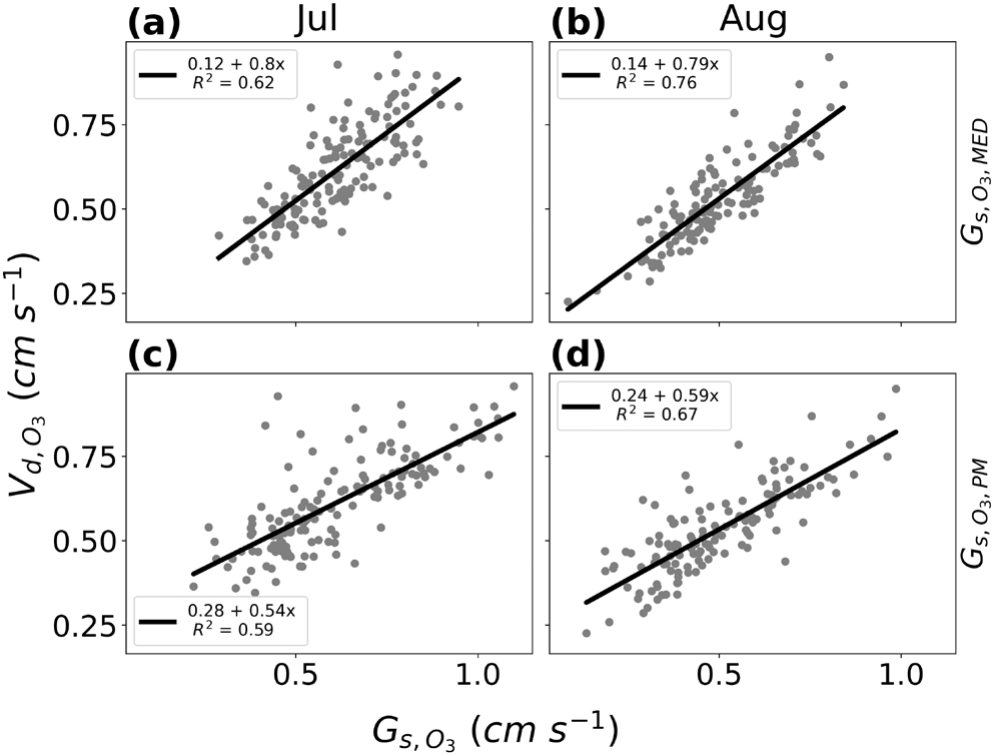
Linear regression between the stomatal conductance to O_3_ ($G_{s,O_{3},\mathrm{MED}}$ and $G_{s,O_{3},\mathrm{PM}}$) and the deposition velocity of $O_{3}$ ($V_{d,O_{3}}$). Plots (a, b) display $G_{s,O_{3},\mathrm{MED}}$ and plots (c, d) display $G_{s,O_{3},\mathrm{PM}}$. Data from July is displayed in plots a and c. Data from August is displayed in plots b and d.

The deposition velocity of O_3_ was highly coupled with both estimates of stomatal conductance, $G_{s,O_{3},\mathrm{MED}}$ and $G_{s,O_{3},\mathrm{PM}}$, through July and August (Figure 4). The highest coupling between the two occurred during August with an *R* ^2^ of 0.76 for $G_{s,O_{3},\mathrm{MED}}$ (Figure 4b) and an *R* ^2^ of 0.67 for $G_{s,O_{3},\mathrm{PM}}$ (Figure 4d). The lowest coupling between stomatal conductance and O_3_ deposition velocity occurred during September with an *R* ^2^ of 0.32 for $G_{s,O_{3},\mathrm{MED}}$ (Figure 10a) and an *R* ^2^ of 0.45 for $G_{s,O_{3},\mathrm{PM}}$ (Figure S10b).

**FIGURE 5:**
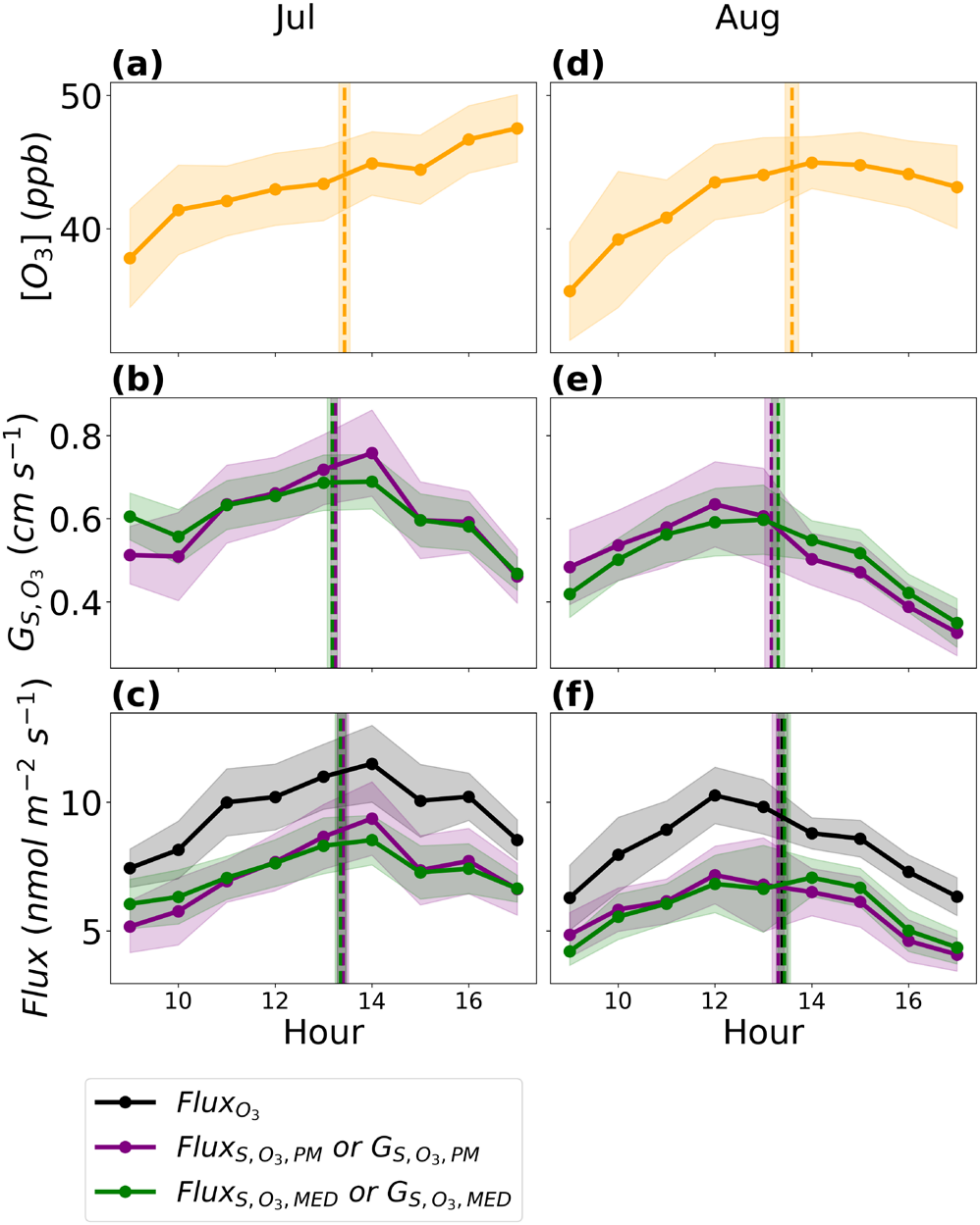
Diurnal patterns of O_3_ concentrations (a, d), the stomatal conductance to O_3_ ($G_{s,O_{3},\mathrm{MED}}$ and $G_{s,O_{3},\mathrm{PM}}$; b, e), the total flux of O_3_ (${\text{Flux}}_{O_{3}}$; c, f), and the stomatal flux of O_3_ (${\text{Flux}}_{s,O_{3},\mathrm{MED}}$ and ${\text{Flux}}_{s,O_{3},\mathrm{PM}}$; c, f). Data from July was used for plots (a—c). Data from August was used for plots (d—f). Dots display the mean. The shaded region display ±2 standard error of the mean (SEM). Only hours when ≥ 5 samples were available were used to calculate the mean and SEM. The vertical dashed lines display the mean diurnal centroid for the month, and the vertical shaded region around the dotted line displays ±2 SEM.

Diurnally, O_3_ concentrations peaked during the afternoon hours (Figure 5a,d) with high afternoon temperatures (Figure S11a,b). During July and August, both estimates of stomatal conductance to O_3_, $G_{s,O_{3},\mathrm{MED}}$ and $G_{s,O_{3},\mathrm{PM}}$, remained higher during the afternoon hours when O_3_ concentrations were at their peak (Figure 5b,e). We did not find reductions in stomatal conductance during high afternoon ${\mathrm{VPD}}_{\text{canopy}‐\mathrm{air}}$ at this maize field (Figure 5b,e and Figure S11c,d). The total flux of O_3_ was higher during the afternoon during July and August (Figure 5c,f). Both estimates of the stomatal flux, ${\text{Flux}}_{s,O_{3},\mathrm{MED}}$ and ${\text{Flux}}_{s,O_{3},\mathrm{PM}}$, were also higher during the afternoon during July and August. The mean diurnal centroid for O_3_ concentrations, $G_{s,O_{3},\mathrm{PM}}$, $G_{s,O_{3},\mathrm{MED}}$, the total flux of O_3_, ${\text{Flux}}_{s,O_{3},\mathrm{PM}}$, and ${\text{Flux}}_{s,O_{3},\mathrm{MED}}$ was between the 13th and 14th hour of the day in July and August (Figure 5). There were limited days that met the criteria for computing diurnal centroids and for calculating a monthly average diurnal centroid. Diurnal centroids for all variables were calculated for 7 days in July and August. During September, the diurnal centroid for all quantities also occurred past the 12th hour of the day (Figure S12). Calculating diurnal centroids using the 9:00–17:00 daylight window did not change these results for July, August, and September.

### 3.3 Accumulated Stomatal Uptake of O_3_ and Exposure to O_3_

**FIGURE 6:**
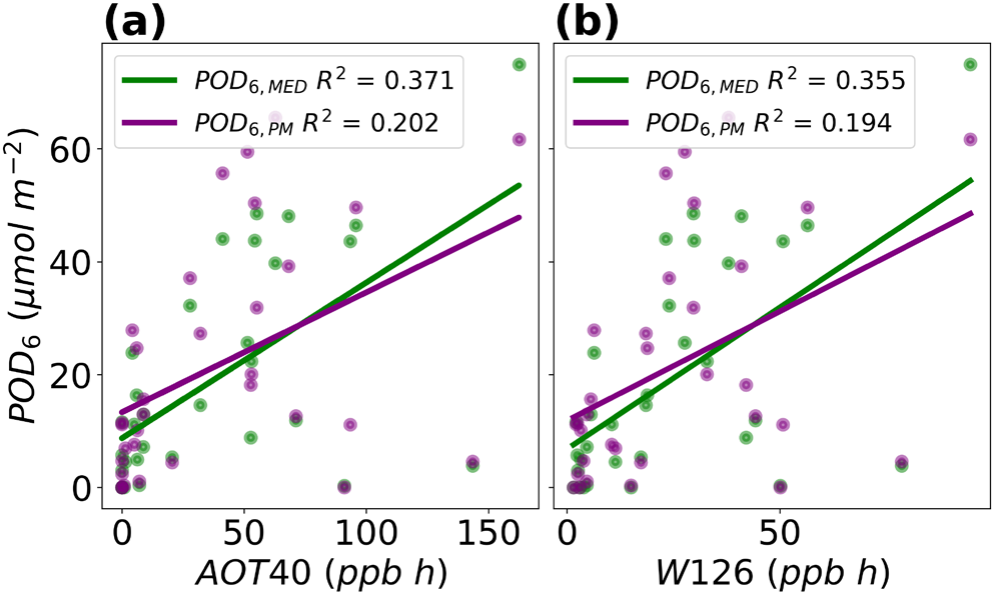
Comparison between daily accumulated exposure to O_3_ using AOT40 (a) and W126 (b) and daily accumulated stomatal flux of O_3_ (POD_6_).

Threshold‐based metrics developed from daily accumulated exposure to O_3_, AOT40 and W126, show a weak linear scaling with the daily phytotoxic dose delivered through the stomata (Figure 6a,b). The *R* ^2^ between POD_6_ and AOT40 is 0.37 for POD_6,MED_ and 0.20 for POD_6,PM_ (Figure 6a). The *R* ^2^ between POD_6_ and W126 is 0.36 for POD_6,MED_ and 0.19 for POD_6,PM_ (Figure 6b). There were a few days when daily accumulated AOT40 and W126 were high but the accumulated POD_6_ showed a large range. For example, for a high daily accumulated AOT40 between 90.96–162.49 ppbh and a high daily accumulated W126 between 50.13–94.79 ppbh, daily accumulated POD_6_ was as low as 0 to as high as 74.69 μmol m^−2^ between the two POD_6_ estimates (Figure 6).

The total accumulated AOT40 over the course of the study period was 2.87 ppm h and the total accumulated W126 was 1.98 ppm h. The total accumulated POD_6_ was 1.16 mmol m^−2^ using ${\text{Flux}}_{s,O_{3},\mathrm{MED}}$ and 0.72 mmol m^−2^ using ${\text{Flux}}_{s,O_{3},\mathrm{PM}}$. The exposure is based on O_3_ concentrations while the stomatal dose requires the estimates of stomatal conductance. Since the quality control filtering for estimates of stomatal conductance is stricter than O_3_ concentrations, there are more observations available to calculate the concentration‐based exposure metrics. Therefore, exposure and stomatal dose were accumulated using a different number of days. The accumulated exposure was calculated using 62 days of data with mean [O_3_] of 37.28 ppb. The stomatal dose was calculated using 34 days of data with mean [O_3_] of 43.40 ppb for POD_MED_ and 32 days with mean [O_3_] of 42.70 ppb using POD_PM_. Therefore, the accumulated dose and exposure should be interpreted as highly conservative estimates, considering that missing observations did not contribute to the calculations but likely added to the actual exposure and dose throughout the study period. Furthermore, the accumulated dose and exposure reported here do not include exposure and dose from the beginning of the growing season before measurements were available.

We tested 15 combinations of quality control filters to estimate POD_6_ with a season‐wide fixed estimation of *G*_0_ and *G*_1_ (Equation 14) and a 10‐day moving estimation of these parameters. The total POD_6,MED_ reduced to 1.01 mmol m^−2^ using a season‐wide estimation of the *G*_0_ and *G*_1_ parameters after filtering out observations collected 36 h after a precipitation event and at times of relative humidity > 80%. This is the quality control filter used to report the accumulated POD_6,MED_ of 1.16 mmol m^−2^ using the 10‐day moving estimation of the *G*_0_ and *G*_1_ parameters. Similarly, accumulated POD_6,MED_ was higher when the *G*_0_ and *G*_1_ parameters are estimated every 10 days compared to a season‐wide estimation of these parameters across most of the 15 combinations of quality control filters (Figure S13). The uncertainty bounds (the minimum and the maximum) for accumulated POD_6,MED_ for the quality control combination that resulted in the highest difference between the two parameter estimation methods is 1.08–1.23 mmol m^−2^ (Figure S13). The mean difference in accumulated POD_6,MED_ between these two parameter estimation methods is 0.07 ± 0.06 mmol m^−2^ across the 15 combinations of quality control filters.

Across all 15 combinations and the two parameter estimation methods, POD_6,MED_ was as low as 0.39 mmol m^−2^ and as high as 1.31 mmol m^−2^ (Figure S13). Below, we report the uncertainty bounds arising from observation filtering for each metric (Figure S13). Since we used a 10‐day moving estimation of the *G*_0_ and *G*_1_ parameters in this study, we continue to report the uncertainty bounds for accumulated POD_6,MED_ arising from observation filtering using the POD_6,MED_ calculated with the 10‐day moving parameter fits. Since we calculated AOT40 and W126 using intersection days with POD_6,MED_ and POD_6,PM_ for each quality control filter, we report two separate uncertainty bounds for accumulated AOT40 and W126 corresponding to each POD_6_ estimate. The exposure metrics will be referred to with a _MED_ or _PM_ subscript according to the dose metric used for intersection days. The uncertainty bounds arising from observation filtering for POD_6,MED_ is 0.45–1.31 mmol m^−2^. The uncertainty bounds for AOT40_MED_ is 1.11–1.78 ppm h, and the uncertainty bounds for W126_MED_ is 0.68–1.18 ppm h. The uncertainty bounds arising from observation filtering for POD_6,PM_ is 0.37–0.81 mmol m^−2^. The uncertainty bounds for AOT10_PM_ is 0.9–1.34 ppm h, and the uncertainty bounds for W126_PM_ is 0.55–0.89 ppm h. The accumulated value for all metrics declines when the days available to calculate the sum of each metric across July and August declines (Figure S13).

The accumulated AOT40_MED_ was 1.73 ppm h, and the accumulated W126_MED_ was 1.12 ppm h when using the same 34 days used to calculate POD_6,MED_ after using the quality control filter that was used in this study (described in Section 2.4). The accumulated AOT40_PM_ was 1.27 ppm h and W126_PM_ was 0.83 ppm h when using the same 32 days used to calculate POD_6,PM_. Unsurprisingly, these accumulated exposure metrics are lower when accumulating exposure over days that intersect with the days used to accumulate POD_6_. However, since the data quality requirements for calculating an exposure metric is lower compared to a dose metric, the AOT40 and W126 reported using the 62 days available to calculate them is closer to the actual exposure experienced by the field. The AOT40 and W126 were reduced to 1.44 and 0.99 ppm h, respectively, when hourly mean [O_3_] were used to calculate the two metrics compared to the half‐hourly [O_3_].

## 4 Discussion

### 4.1 Stomatal Conductance Drives Tropospheric O_3_ Flux Over a Maize Agriultural Field and Explains Diurnal Patterns in Total O_3_ Flux

Crop ecophysiology was the dominant driver of O_3_ flux to this maize agricultural field during times of peak LAI and canopy height (July and August). During this time, the total flux of O_3_ was largely made up of the stomatal flux, and environmental conditions during the afternoon favored both high stomatal conductance and high O_3_ concentrations creating an ideal situation for stomatal uptake during heightened O_3_ exposure. Evapotranspiration (which was dominantly transpiration), CO_2_ assimilation, and stomatal conductance remained high when air temperature and ${\mathrm{VPD}}_{\text{canopy}‐\mathrm{air}}$ were high. The photosynthetic and stomatal response of the C_4_ maize crop to the hot and humid environment created temporal synchrony between high O_3_ concentrations, high stomatal conductance, and hence high stomatal uptake of O_3_. This is in direct contrast to many of the existing findings comparing the diurnal and seasonal course of O_3_ concentrations and the stomatal O_3_ flux‐based, in part, on findings in Mediterranean ecosystems. In these cases, peak O_3_ exposure is often not aligned with peak O_3_ uptake due to stomatal regulation (Heath et al. 2009; Fares, Goldstein, and Loreto 2010; Fares, McKay, et al. 2010).

The monthly average stomatal fraction of the total flux of O_3_ ranged from 0.72–0.75 for July and August. The non‐stomatal flux is likely due to the destruction of O_3_ on leaf cuticles, which has been modeled as an active non‐stomatal sink in maize during the growing season (Stella et al. 2011; Stella, Personne, et al. 2013). The canopy structure was highly developed throughout most of the study period, meaning maximum canopy height and LAI were reached early in the study period by mid‐July. This tall and dense canopy likely increases the resistance to within‐canopy transport of O_3_ to the soil, rendering within‐canopy transport a negligible non‐stomatal sink. Soil uptake was likely a part of the non‐stomatal flux in canopy gaps with exposed bare soil. It is also possible that within‐canopy transport to the soil and soil uptake, both of which have been suggested as significant non‐stomatal sinks in maize fields (Van Pul and Jacobs 1994), are more important during the beginning of the growing season while the canopy is still developing and is less dense. We found the lowest coupling between O_3_ deposition velocity and stomatal conductance and the lowest stomatal fraction of total O_3_ flux during September. This indicates that the non‐stomatal flux contributed highly to the variability of the total O_3_ flux during September.

### 4.2 The Field Likely Experienced Critical Levels of Phytotoxic Stomatal Dose of O_3_ Even When O_3_ Concentrations Were Below 70 ppb

Daily AOT40 and W126 showed a weak linear scaling with the daily phytotoxic stomatal dose. Critical levels of AOT40 at 8.67 ppm h and 13.9 ppm h and POD_6_ at 1.17 mmol m^−2^ LA have been reported for maize (Mills et al. 2007; Peng et al. 2019). At this level of AOT40 and POD_6_, the maize crop was reported to suffer 5% yield losses (Mills et al. 2007; Peng et al. 2019). Our estimate of accumulated AOT40 over 62 days, 2.87 ppm h, does not reach the reported critical levels, but it is important to note that our estimate is conservative. It is possible that our field experienced critical levels of O_3_ exposure with AOT40 accumulated over the entire season. It is also important to note that we accumulated AOT40 using half‐hourly observations of O_3_ concentrations. AOT40 used for calculating critical levels is often calculated using hourly mean O_3_ concentrations in the literature (Mills et al. 2007). After recalculating AOT40 with hourly mean [O_3_], we found a lower accumulated AOT40 of 1.45 ppm h.

Even though our estimates of accumulated exposure are lower than reported critical levels, our estimates of POD_6_, 1.16 mmol m^−2^ (${\text{Flux}}_{s,O_{3},\mathrm{MED}}$) and 0.72 mmol m^−2^ (${\text{Flux}}_{s,O_{3},\mathrm{PM}}$) are close to the critical levels previously reported. However, it is important to note that reported critical levels of POD_6_ for maize use a stomatal conductance model parametrized with leaf‐level measurements to quantify stomatal O_3_ uptake (Peng et al. 2019), and stomatal uptake quantified at the field‐scale with eddy covariance measurements does not directly compare to critical levels reported in the literature. Therefore, it is currently unclear how critical the field‐scale POD_6_ values that we report are. The time period used to calculate POD_6_ in this study consisted of a near stable LAI of around 4 m^2^ m^−2^. Therefore, it is likely that as total ozone uptake per unit area increased, phytotoxicity increased as well. Future studies that quantify POD_6_ from the eddy covariance technique across the full season should take changing LAI—from early to late crop development—into account. It is likely that the field experienced POD_6_ that exceeded previously reported critical levels if accumulation was not limited to July and August. Our findings echo the suggestion of Cieslik (2004) that metrics like AOT40 do not hold much value where O_3_ concentrations above 40 ppb are low, but the vegetation is highly photosynthetically active, resulting in a high stomatal dose. While uncertainties remain in applying dose–response relationships, reported critical levels are our only existing frame of reference to mark dangerous levels of O_3_. Long‐term monitoring of co‐located O_3_, CO_2_ and H_2_0 fluxes, along with yields, will reveal a field‐specific sensitivity of yields to the phytotoxic stomatal dose. O_3_ flux observations largely rely on chemiluminescence‐based instruments, which can be difficult to maintain in the field, but we show that the NASA ROZE UV‐absorption‐based instrument is capable of measuring O_3_ flux, and it will be crucial in long‐term monitoring.

The temporal synchrony between exposure and dose and the maize crop's high stomatal uptake during the growing season could partly explain the higher O_3_ induced yield losses that have been reported for maize and the higher sensitivity of maize yield losses to both W126 and AOT40 compared to soybean (McGrath et al. 2015). However, estimates of O_3_ stomatal uptake over soybean fields in the same region are required to confirm this considering the temporal synchrony could be observed over well‐watered soybean in continental humid climates. Global environmental change can differentially impact the components of air pollution‐crop yield dynamics. While regulations can somewhat control ambient O_3_ concentrations over the available arable land (McGrath et al. 2015), exceptional events such as increasing wildfire smoke and other uncertain contributors such as intercontinental transport could impact O_3_ exposure. Increases in atmospheric [CO_2_] can reduce the stomatal conductance of maize crops (Leakey et al. 2006) possibly decoupling exposure and uptake at maize fields in the future as atmospheric [CO_2_] continues to increase. The uncertainty in the future availability of arable land coupled with the facts that current statistical models already suggest a negative impact of O_3_ exposure on yields and that global environmental change will impact O_3_ concentrations and stomatal conductance through different mechanisms warrants increased flux‐based monitoring of the field scale phytotoxic dose of O_3_ to monitor and assess the risk that O_3_ presents to crop yields.

### 4.3 Increased O_3_ Flux Observation Will Help Capture the Variability of O_3_ Dry Deposition Over Agricultural Land

Crop ecophysiology in our maize agricultural field created a significant sink of O_3_ during peak leaf area and canopy height. The monthly mean total flux ranged from 5.08 to 9.76 nmol m^−2^ s^−1^, and the monthly mean deposition velocity ranged from 0.33 to 0.61 cm s^−1^. The range of both deposition velocity and total O_3_ flux are in the range previously reported for maize fields during peak green leaf area in France although growing season deposition velocities as low as 0.2 cm s^−1^ have been reported in Oak Ridge, TN, Argonne, IL, and State College, PA (Meyers and Hicks 1988; Stella et al. 2011; Stella, Personne, et al. 2013). Our observed monthly mean total O_3_ flux during July is higher than the July monthly means observed over potato fields, which fall within 6–8 nmol m^−2^ s^−1^, and it is slightly lower than the June monthly mean observed over onion and beet crops which falls within 10–12 nmol m^−2^ s^−1^ (Gerosa et al. 2007; Coyle et al. 2009; Hardacre et al. 2015). Hardacre et al. (2015) showed an overestimation of monthly average O_3_ flux over maize by global scale chemistry climate models, but our observed July and August monthly average O_3_ fluxes are higher than the monthly averages of the maize flux observations used in the comparison and are on the higher end of model estimates which fall within 4–8 nmol m^−2^ s^−1^ during July and August.

The small number of short‐term measurements of agricultural O_3_ flux have revealed that total and stomatal flux and their seasonality across different crop fields can be highly variable due to aridity, management practices such as irrigation, crop structural impacts on how much bare soil is exposed, and non‐stomatal dry deposition processes such as cuticular destruction of O_3_ (Cieslik 2004, 2009; Gerosa et al. 2007; Potier et al. 2015, 2017). Adding to the existing sparse measurements, our observations of total O_3_ flux and estimates of partitioned stomatal flux over maize contribute a case where we did not observe a significant non‐stomatal component during times of high GPP. This suggests a need for increased long‐term O_3_ flux observations over agricultural areas that can help capture the variability of O_3_ dry deposition over different cropping types in various climates.

## 5 Conclusion

Phytotoxic air pollutants like tropospheric O_3_ cause reductions in crop yields. O_3_ concentrations are routinely monitored, but they cannot alone quantify the stomatal dose of O_3_. While there are existing methods to calculate the stomatal dose based on stomatal conductance models that only require meteorological conditions, flux‐based estimates are calculated using the surface‐atmosphere exchange of the very gasses that define the conductance. In the face of global environmental change, we need co‐located monitoring of O_3_, CO_2_, and H_2_O flux and crop yields. Here, we monitored O_3_ flux using a custom UV‐absorption based instrument along with CO_2_ and H_2_O flux at a maize agricultural field during the growing season and asked:

1. How does maize ecophysiology impact the stomatal dose of O_3_ through the peak and end of the growing season over an agricultural field in the continental humid, midwestern region of the United States?
2. How does the phytotoxic stomatal dose of O_3_ compare with exposure‐based metrics calculated from O_3_ concentrations at this field?

To answer Question 1, we found that the photosynthetic and stomatal response of the maize crop to the hot and humid environment created temporal synchrony between high O_3_ concentrations and high stomatal conductance. The crop did not substantially reduce stomatal conductance in response to high afternoon VPD when O_3_ concentrations were at their peak, resulting in a continued stomatal dose during peak concentrations. We also found that the stomatal flux of O_3_ made up the majority of the total flux of O_3_ at this field when GPP, green leaf area, and stomatal conductance were high.

To answer Question 2, the daily accumulated phytotoxic stomatal dose of O_3_ scaled poorly with daily accumulated exposure metrics such as W126 and AOT40 at this field. The field likely experienced an accumulated stomatal dose of O_3_ that exceeded previously reported critical levels at which 5% yield reductions were reported for maize. Studies of ozone exposure in maize have demonstrated that yield loses scale with the stomatal uptake of O_3_ (Peng et al. 2019). However, phytotoxicity is also determined by antioxidant capacity. If antioxidant capacity does not increase with increasing leaf area, then increased canopy scale stomatal uptake of ozone through increases in leaf area can be expected to lead to increases in phytotoxicity. The exposure‐dose dynamics at this maize field in a continental humid environment did not present diurnal asynchrony between O_3_ exposure and stomatal O_3_ flux during the study period, meaning stomatal regulation did not protect the maize crop from peak O_3_ exposure. Due to maize's high LAI and high optimum temperatures for photosynthesis along with sustained stomatal uptake of O_3_ during peak O_3_ concentrations, the field likely experienced a critical level of stomatal O_3_ dose even when O_3_ concentrations did not go too far above 40 ppb and remained below 70 ppb. NASA ROZE can serve as an effective UV‐absorption‐based instrument to continue monitoring of O_3_ flux at eddy covariance towers that also measure CO_2_ and H_2_O flux to ensure that we will have proper spatiotemporal coverage of agricultural land for flux‐based yield loss predictions and monitoring the phytotoxic dose of O_3_.

## Author Contributions

**Reem Hannun:** conceptualization, methodology, data curation, investigation, formal analysis, resources, software, writing – review and editing. **Elizabeth A. Ainsworth:** conceptualization, resources, writing – review and editing, project administration. **Carl J. Bernacchi:** data curation, resources, writing – review and editing. **Anam M. Khan:** conceptualization, methodology, formal analysis, investigation, writing – original draft, data curation, visualization, writing – review and editing. **Taylor Pederson:** writing – review and editing, data curation, resources. **Lun Gao:** conceptualization, data curation, writing – review and editing, investigation, methodology. **Paul C. Stoy:** funding acquisition, conceptualization, methodology, supervision, resources, writing – review and editing. **Kaiyu Guan:** writing – review and editing.

## Funding

This work was supported by the National Science Foundation, 2106012. University of Wisconsin‐Madison Office of Vice Chancellor for Research and Graduate Education with funding from the Wisconsin Alumni Research Foundation.

## Conflicts of Interest

The authors declare no conflicts of interest.

## Supporting information


**Figure S1:** A diagram describing the estimation of stomatal conductance and stomatal O_3_ flux. Detailed methods are described in the paper accompanying this supplementary figure. The scatter plot shows stomatal conductance estimated from Equation (11) (gray dots) and Equation (14) (green dots) of the paper on the *y*‐axis. The *x*‐axis displays values of the terms from the stomatal conductance model described in Equation (14) of the paper accompanying this supplementary figure.
**Figure S2:** O_3_ concentrations (a), deposition velocity of O_3_ (*V*_*d*_, O_3_; b), stomatal conduc tance to O_3_ (*G*_*s*_, O_3_, MED and *G*_*s*_, O_3_, PM; b), total flux of O_3_ (Flux O_3_; c), and stomatal flux of O_3_ (Flux_*s*_, O_3_, MED and Flux_*s*_, O_3_, PM; c). Gray shaded areas display days with precipitation and 36 h after precipitation.
**Figure S3:** The data available for analysis after all data filters were applied as a percent of total data available without the data filters (a). The data available for analysis after each type of data filter was applied as a percent of total data available without the data filter (b). Data filters are described in Sections 2.2, 2.4 of the paper associated with this supplementary figure. The percentage is displayed for each day. For subplot a, the color created by the overlap in the two colors is used to display the overlap in percent data available to calculate *G*_*s*_, PM and *G*_*s*_, MED after all data filters are applied.
**Figure S4:** The data available for analysis after all data filters were applied as a percent of total data available without the data filters (a). The data available for analysis after each type of data filter was applied as a percent of total data available without the data filter (b). Data filters are described in Sections 2.2, 2.4 of the paper associated with this supplementary figure. The percentage is displayed for each hour of the day. For subplot a, the color created by the overlap in the two colors is used to display the overlap in percent data available to calculate *G*_*s*_, PM and *G*_*s*_, MED after all data filters are applied.
**Figure S5:** Leaf area index (LAI; a), and canopy height (b) at the eddy covariance site in central Illinois near Champaign, IL.
**Figure S6:** Distribution of the stomatal conductance to O_3_ (*G*_*s*_, O_3_) with increasing VPDcanopy−air and changes in atmospheric stability (*r*‐*d*/*L*). The boxes display the interquartile range (IQR) with the median marked with a horizontal line inside the box. The whiskers extend 1.5 IQR on either side of the box.
**Figure S7:** Distribution of the stomatal conductance to O_3_ (*G*_*s*_, O_3_) with increasing VPDcanopy−air and changes in wind direction. The boxes display the interquartile range (IQR) with the median marked with a horizontal line inside the box. The whiskers extend 1.5 IQR on either side of the box.
**Figure S8:** Distribution of the stomatal conductance to O_3_ (*G*_*s*_, O_3_) with changes in air tempera ture (*T*_air_) and incoming shortwave radiation (SW_in_). Dots display the mean and the error bars display the ±2 standard error of the mean.
**Figure S9:** Distribution of the stomatal conductance to O_3_ (*G*_*s*_, O_3_) with changes in canopy‐air VPD (VPDcanopy−air) and incoming shortwave radiation (SW_in_). Dots display the mean and the error bars display the ±2 standard error of the mean.
**Figure S10:** Linear regression between the stomatal conductance to O_3_ (*G*_*s*_, O_3_, MED and *G*_*s*_, O_3_, PM) and the deposition velocity of O_3_ (*V*_*d*_, O_3_). Plot a displays *G*_*s*_, O_3_, MED, and plot b displays *G*_*s*_, O_3_, PM. Data from September was used for the regression displayed in plots a and b.
**Figure S11:** Diurnal patterns in air temperature (*T*_air_; a, b), canopy‐ air vapor pressure deficit (V PDcanopy−air; c, d), and incoming shortwave radiation (SW_in_; e, f). Dots display the mean calculated at each hour during a given month. The shaded region displays ±2 standard error of the mean (SEM) for each hour. Only hours when ≥ 5 samples were available were used to calculate the mean and SEM.
**Figure S12:** Diurnal patterns of O_3_ concentrations (a), the stomatal conductance to O_3_ (*G*_*s*_, O_3_, MED and *G*_*s*_, O_3_, PM; b), the total flux of O_3_ (Flux O_3_; c), and the stomatal flux of O_3_ (Flux_*s*_, O_3_, MED and Flux_*s*_, O_3_, PM; c). Data from September was used for plots a–c. Dots dis play the mean. The shaded region display ±2 standard error of the mean (SEM). Only hours when ≥ 5 samples were available were used to calculate the mean and SEM. The vertical dashed lines display the mean diurnal centroid for the month, and the vertical shaded region around the dotted line displays ±2 SEM.
**Figure S13:** Changes in POD6, AOT40, and W126 with changes in the filter that removes observations collected immediately after a precipitation event and at times of high relative humidity. The metrics were calculated after optimizing stomatal conductance estimates with each subset of observations specific to a given relative humidity and post precipitation filter. The colors display the changing values of the metric with changes in observations removed by each relative humidity filter. The *x*‐axis displays the post precipitation filter used to remove observations for calculating the metrics. The last row displays the change in the number of days available to calculate each metric as further filtering is applied. The dotted lines in the top left plot display the estimates of POD_6_, MED using a season‐wide fixed estimate of the parameters used to calculate *G*_*s*_, O_3_, MED. The column labeled “MED” displays results for all metrics calculated on days when *G*_*s*_, O_3_, MED was available. The column labeled “PM” displays results for all metrics calculated on days when *G*_*s*_, O_3_, PM was available.

**Data S1:** Data S1 contains the data from Table 1 of this manuscript.

## Data Availability

The ozone data used in this analysis is available on Zenodo at https://doi.org/10.5281/zenodo.16756683. All other eddy covariance flux tower data used in this analysis will be available on AmeriFlux at http://doi.org/10.17190/AMF/1246036.
