# Supplementary material for: Maize Crops Face High Stomatal Uptake During High Exposure to Ozone in an Agroecosystem in the United States Corn Belt
Source: Glob Chang Biol. 2026 Jun 5;32(6):e70952. doi: 10.1111/gcb.70952 (PMC13240603; doi:10.1111/gcb.70952)
Supplement: Supplementary file 1 — Figure S1: A diagram describing the estimation of stomatal conductance and stomatal O3 flux. Detailed methods are described in the paper accompanying this supplementary figure. The scatter plot shows stomatal conductance estimated from Equation (11) (gray dots) and Equation (14) (green dots) of the paper on the y‐axis. The x‐axis displays values of the terms from the stomatal conductance model described in Equation (14) of the paper accompanying this supplementary figure. Figure S2: O3 concentrations (a), deposition velocity of O3 (V d , O3; b), stomatal conduc tance to O3 (G s , O3, MED and G s , O3, PM; b), total flux of O3 (Flux O3; c), and stomatal flux of O3 (Flux s , O3, MED and Flux s , O3, PM; c). Gray shaded areas display days with precipitation and 36 h after precipitation. Figure S3: The data available for analysis after all data filters were applied as a percent of total data available without the data filters (a). The data available for analysis after each type of data filter was applied as a percent of total data available without the data filter (b). Data filters are described in Sections 2.2, 2.4 of the paper associated with this supplementary figure. The percentage is displayed for each day. For subplot a, the color created by the overlap in the two colors is used to display the overlap in percent data available to calculate G s , PM and G s , MED after all data filters are applied. Figure S4: The data available for analysis after all data filters were applied as a percent of total data available without the data filters (a). The data available for analysis after each type of data filter was applied as a percent of total data available without the data filter (b). Data filters are described in Sections 2.2, 2.4 of the paper associated with this supplementary figure. The percentage is displayed for each hour of the day. For subplot a, the color created by the overlap in the two colors is used to display the overlap in percent data available to calcul [file GCB-32-e70952-s001.pdf]

Supplementary material for the submitted manuscript by Khan et al.: Maize crops face high stomatal uptake during high exposure to ozone in an agroecosystem in the United States Corn Belt

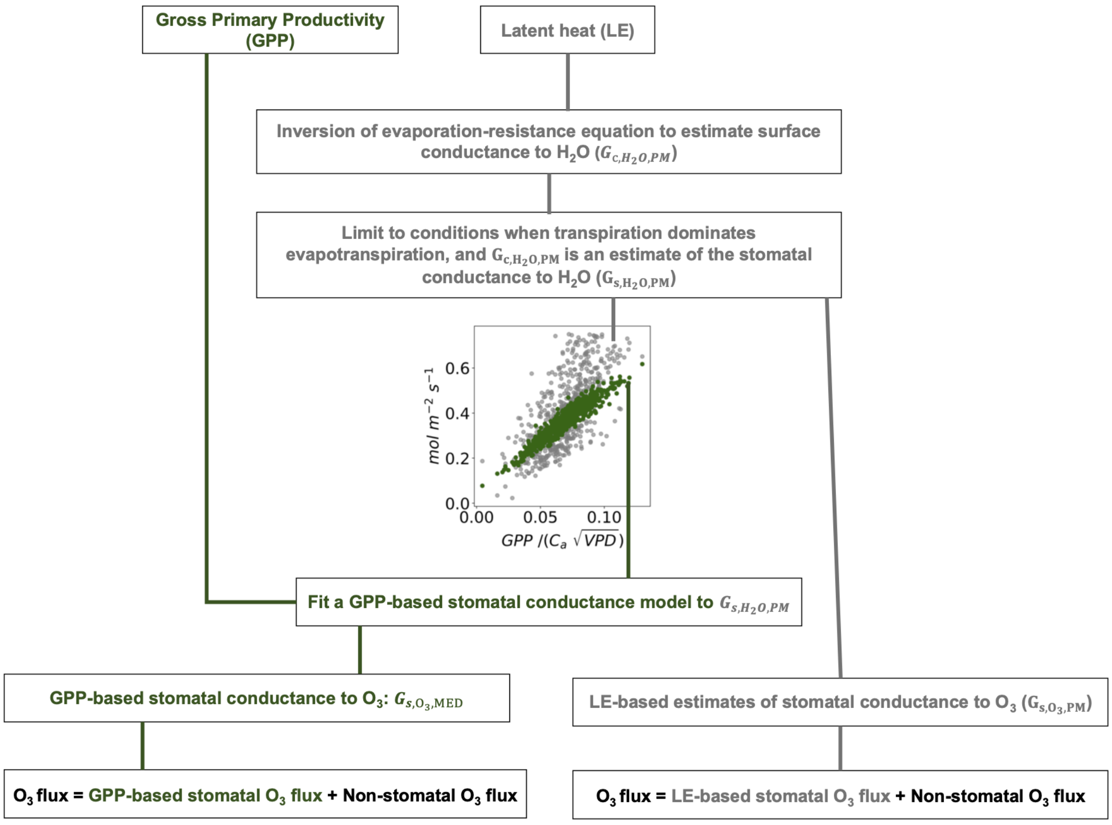

Figure S1: A diagram describing the estimation of stomatal conductance and stomatal O_3 flux. Detailed methods are described in the paper accompanying this supplementary figure. The scatter plot shows stomatal conductance estimated from equation 11 (gray dots) and equation 14 (green dots) of the paper on the y-axis. The x-axis displays values of the terms from the stomatal conductance model described in equation 14 of the paper accompanying this supplementary figure.

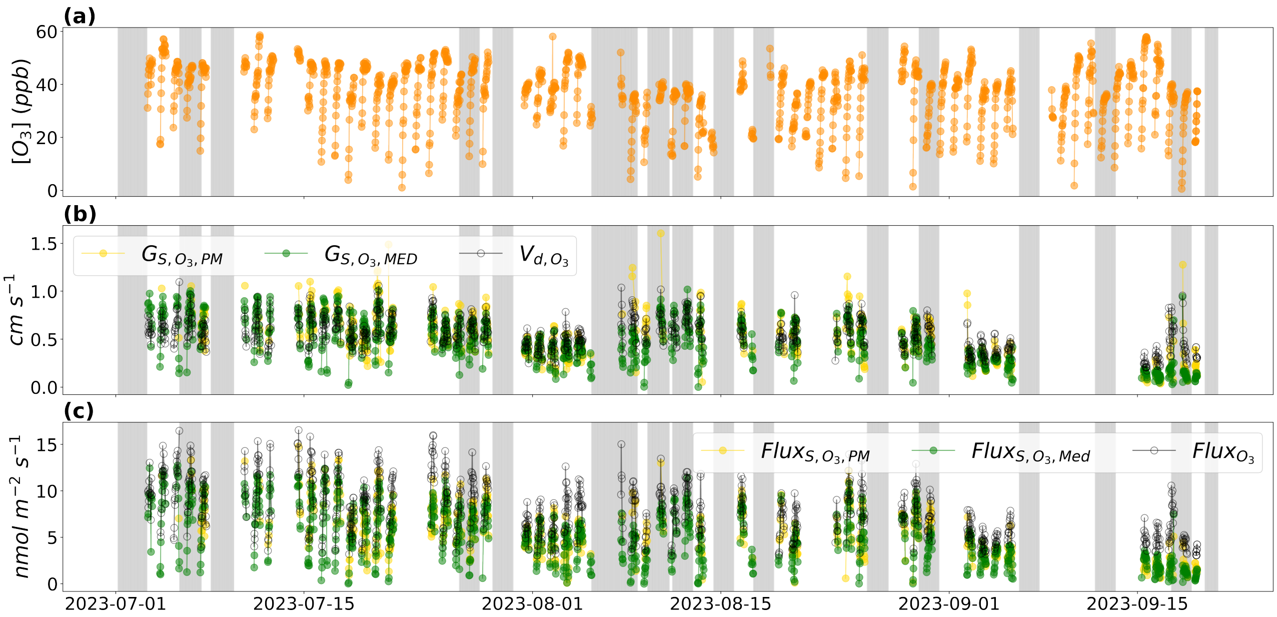

Figure S2: O_3 concentrations (a), deposition velocity of O_3 (V_{d,O_3} ; b), stomatal conductance to O_3 ($G_{s,O3,MED}$ and $G_{s,O_3,PM}$; b), total flux of O_3 ($Flux_{O_3}$; c), and stomatal flux of O_3 ($Flux_{s,O_3,MED}$ and $Flux_{s,O_3,PM}$; c). Gray shaded areas display days with precipitation and 36 hours after precipitation.

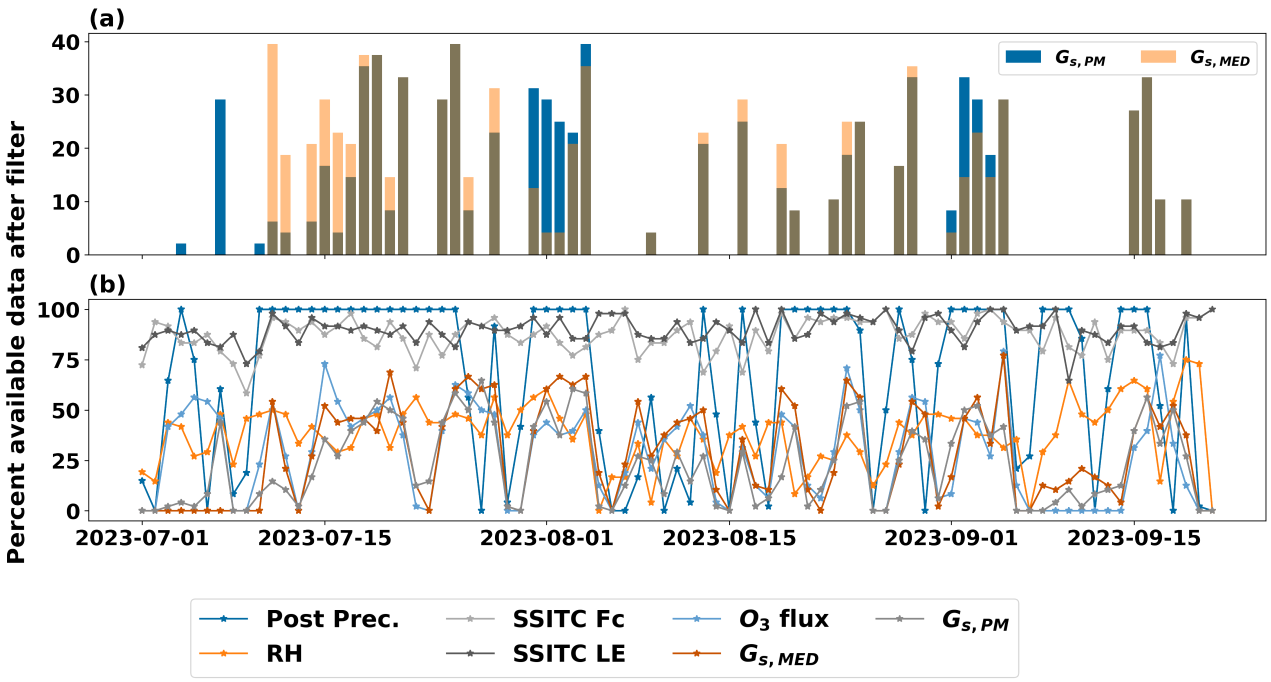

Figure S3: The data available for analysis after all data filters were applied as a percent of total data available without the data filters (a). The data available for analysis after each type of data filter was applied as a percent of total data available without the data filter (b). Data filters are described in sections 2.2 - 2.4 of the paper associated with this supplementary figure. The percentage is displayed for each day. For subplot a, the color created by the overlap in the two colors is used to display the overlap in percent data available to calculate $G_{s,PM}$ and $G_{s,MED}$ after all data filters are applied.

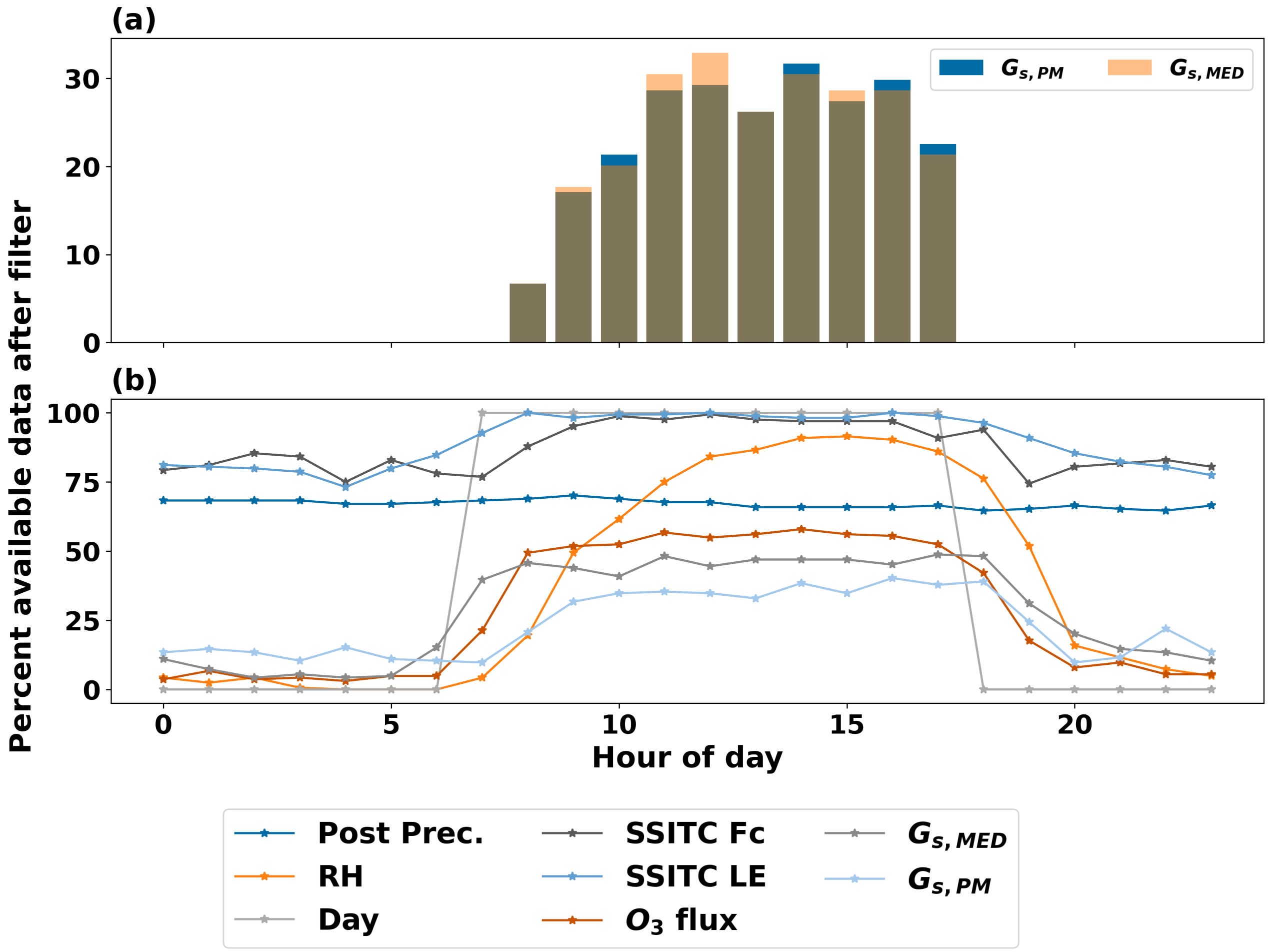

Figure S4: The data available for analysis after all data filters were applied as a percent of total data available without the data filters (a). The data available for analysis after each type of data filter was applied as a percent of total data available without the data filter (b). Data filters are described in sections 2.2 - 2.4 of the paper associated with this supplementary figure. The percentage is displayed for each hour of the day. For subplot a, the color created by the overlap in the two colors is used to display the overlap in percent data available to calculate $G_{s,PM}$ and $G_{s,MED}$ after all data filters are applied.

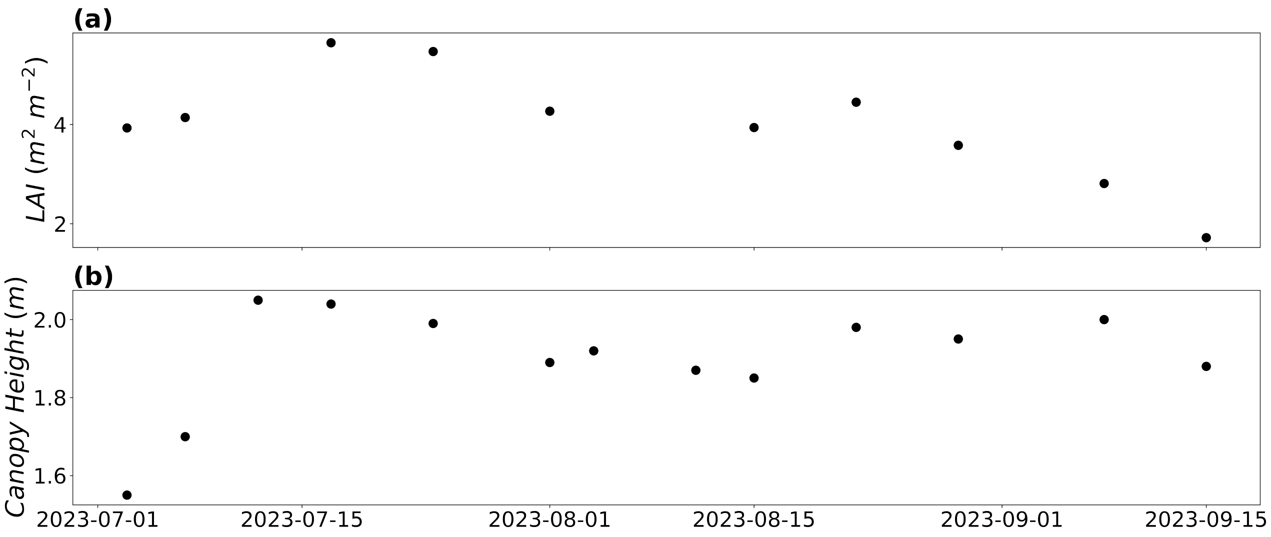

Figure S5: Leaf area index (LAI; a), and canopy height (b) at the eddy covariance site in central Illinois near Champaign, IL.

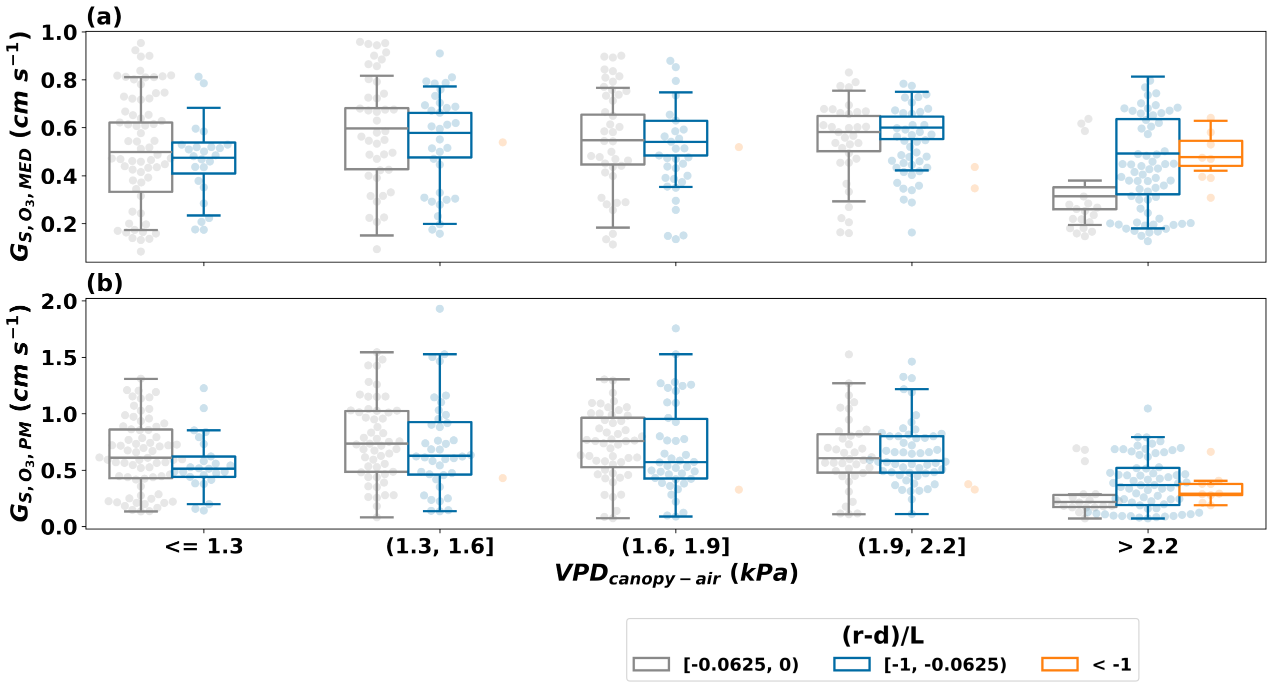

Figure S6: Distribution of the stomatal conductance to O_3 (G_{s,O_3}) with increasing $VPD_{canopy-air}$ and changes in atmospheric stability (r-d/L). The boxes display the interquartile range (IQR) with the median marked with a horizontal line inside the box. The whiskers extend 1.5 IQR on either side of the box.

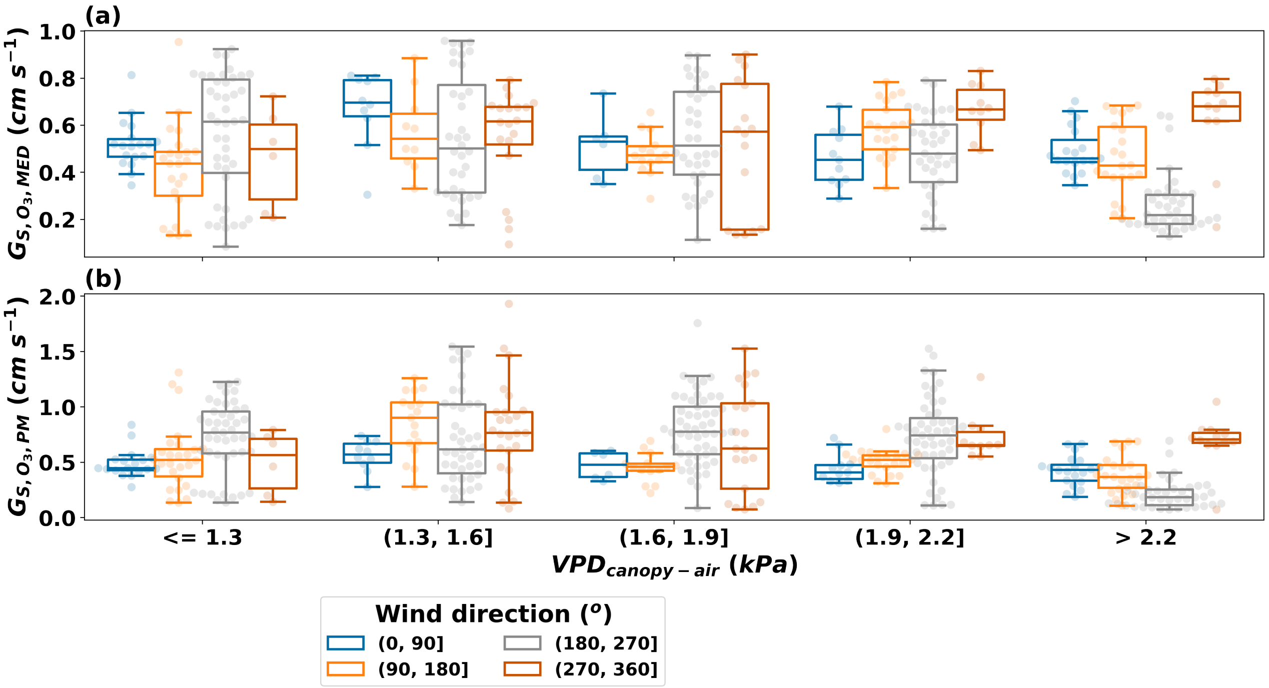

Figure S7: Distribution of the stomatal conductance to O_3 (G_{s,O_3}) with increasing $VPD_{canopy-air}$ and changes in wind direction. The boxes display the interquartile range (IQR) with the median marked with a horizontal line inside the box. The whiskers extend 1.5 IQR on either side of the box.

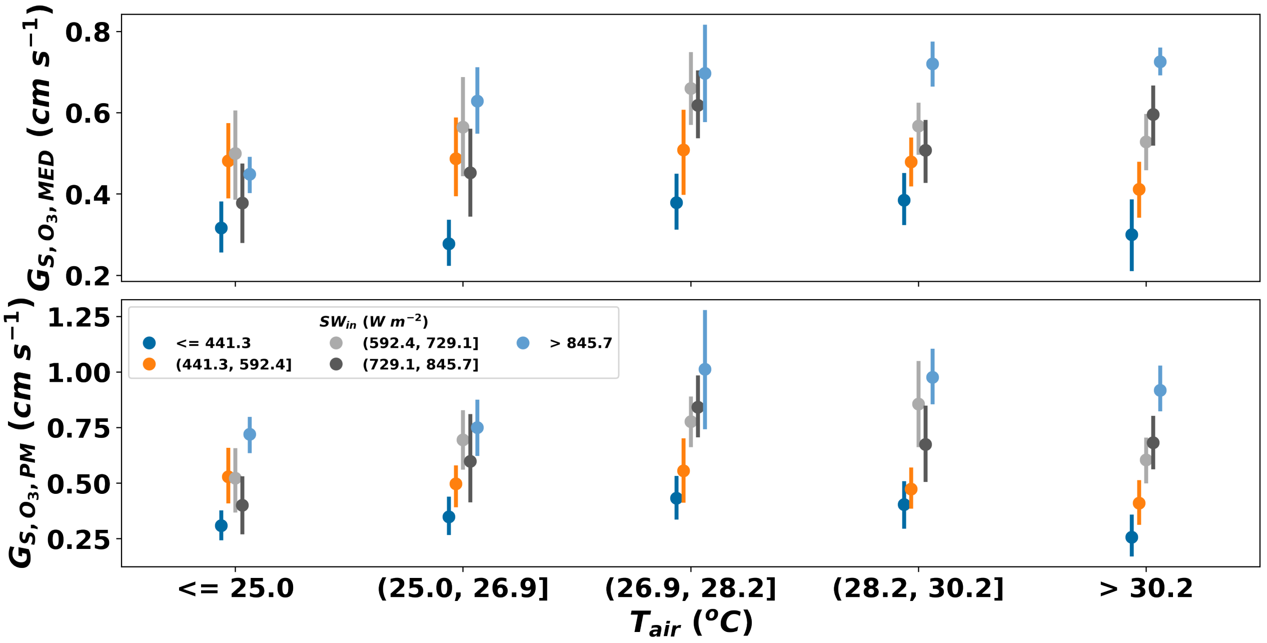

Figure S8: Distribution of the stomatal conductance to O_3 (G_{s,O_3}) with changes in air temperature (T_{air}) and incoming shortwave radiation (SW_{in}). Dots display the mean and the error bars display the $\pm$ 2 standard error of the mean.

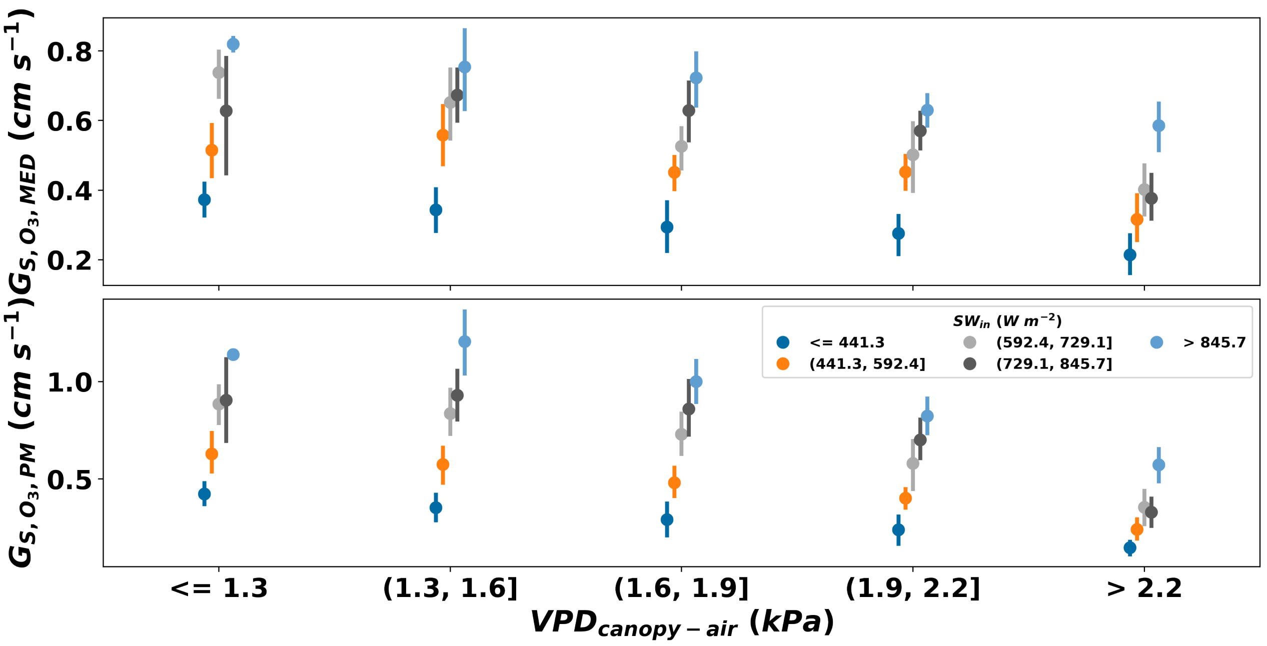

Figure S9: Distribution of the stomatal conductance to O_3 (G_{s,O_3}) with changes in canopy-air VPD ($VPD_{canopy-air}$) and incoming shortwave radiation (SW_{in}). Dots display the mean and the error bars display the $\pm$ 2 standard error of the mean.

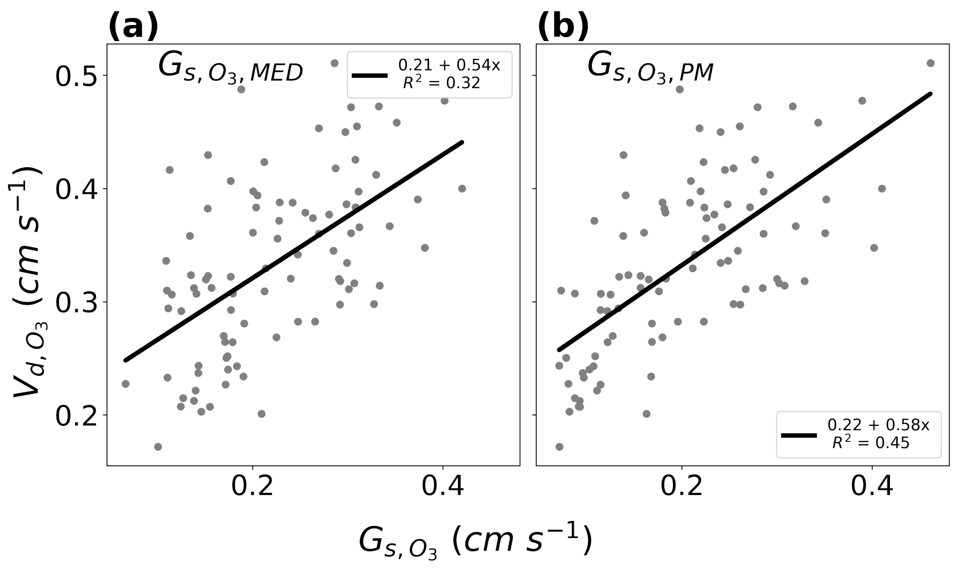

Figure S10: Linear regression between the stomatal conductance to O_3 ($G_{s,O_3,MED}$ and $G_{s,O_3,PM}$) and the deposition velocity of O_3 (V_{d,O_3}). Plot a displays $G_{s,O_3,MED}$, and plot b displays $G_{s,O_3,PM}$. Data from September was used for the regression displayed in plots a and b.

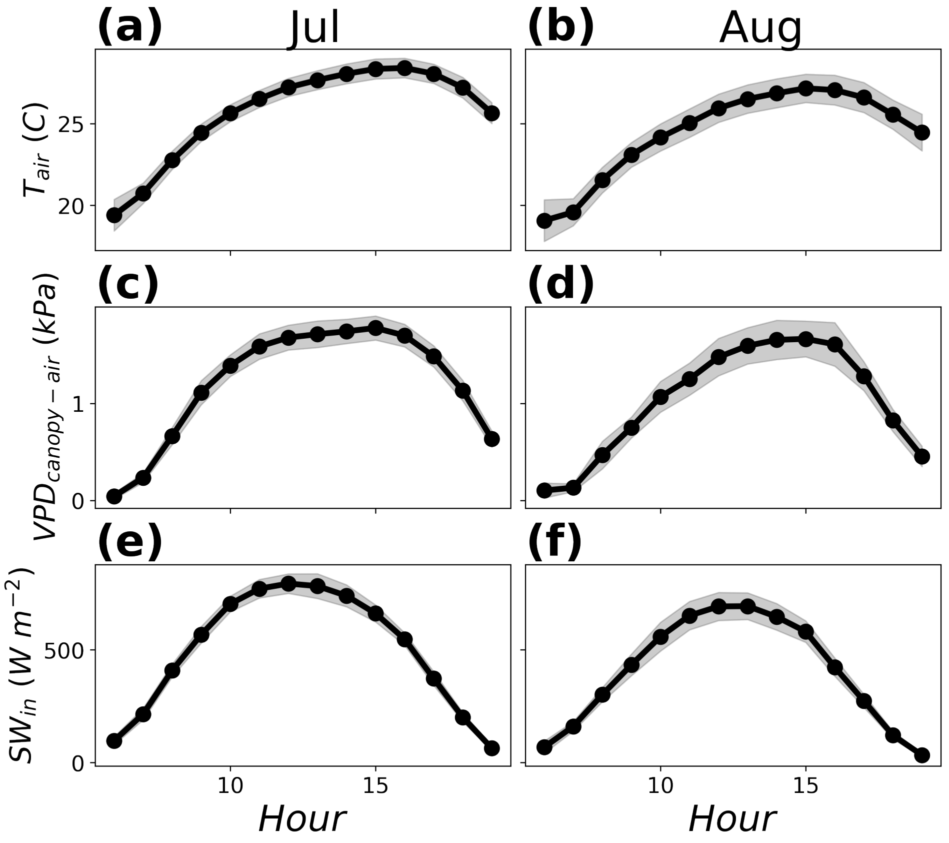

Figure S11: Diurnal patterns in air temperature (T_{air}; a-b), canopy - air vapor pressure deficit ($VPD_{canopy-air}$; c-d), and incoming shortwave radiation (SW_{in}; e-f). Dots display the mean calculated at each hour during a given month. The shaded region displays $\pm$ 2 standard error of the mean (SEM) for each hour. Only hours when $\geq$ 5 samples were available were used to calculate the mean and SEM..

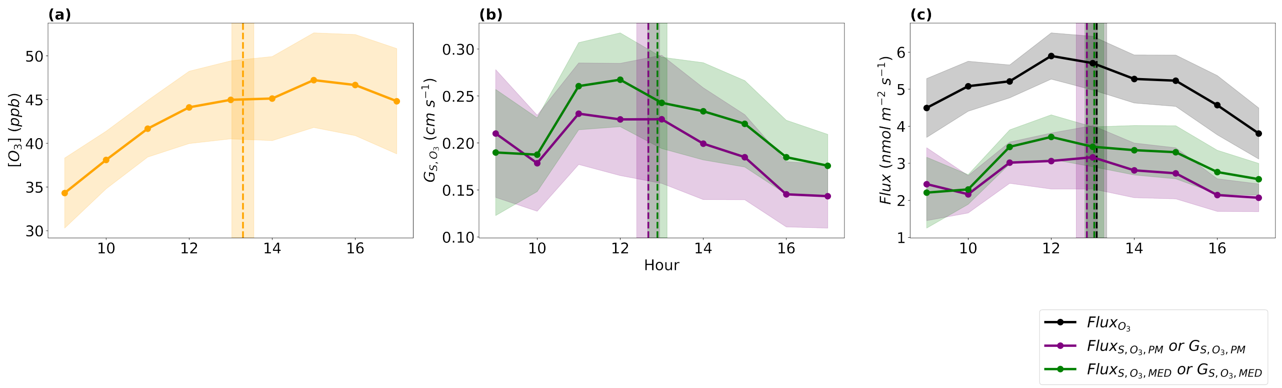

Figure S12: Diurnal patterns of O_3 concentrations (a), the stomatal conductance to O_3 ($G_{s,O_3,MED}$ and $G_{s,O_3,PM}$; b), the total flux of O_3 ($Flux_{O_3}$; c), and the stomatal flux of O_3 ($Flux_{s,O_3,MED}$ and $Flux_{s,O_3,PM}$; c). Data from September was used for plots a - c. Dots display the mean. The shaded region display $\pm$ 2 standard error of the mean (SEM). Only hours when $\geq$ 5 samples were available were used to calculate the mean and SEM. The vertical dashed lines display the mean diurnal centroid for the month, and the vertical shaded region around the dotted line displays $\pm$ 2 SEM.

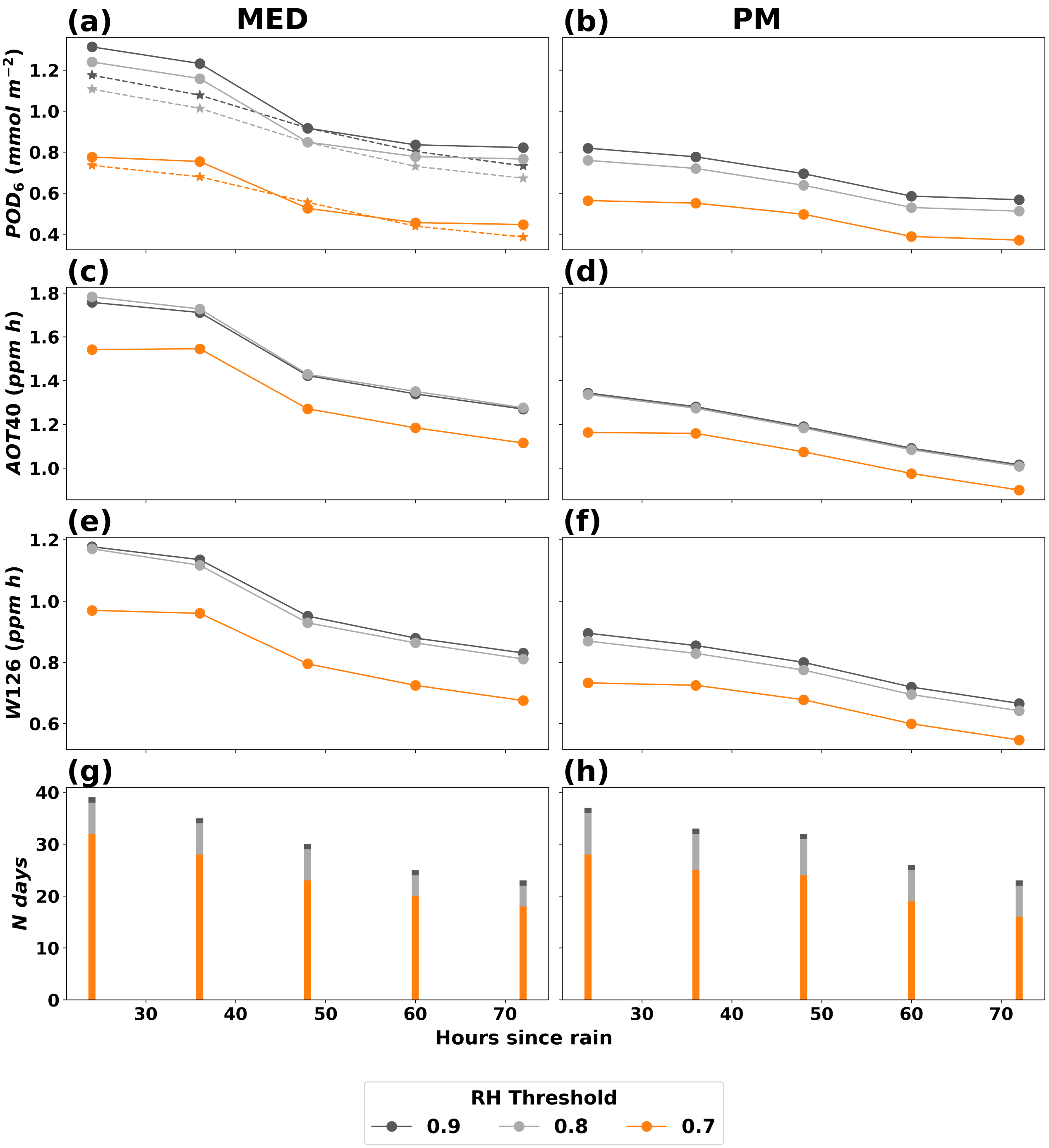

Figure S13: Changes in POD_6, AOT40, and W126 with changes in the filter that removes observations collected immediately after a precipitation event and at times of high relative humidity. The metrics were calculated after optimizing stomatal conductance estimates with each subset of observations specific to a given relative humidity and post precipitation filter. The colors display the changing values of the metric with changes in observations removed by each relative humidity filter. The x-axis displays the post precipitation filter used to remove observations for calculating the metrics. The last row displays the change in the number of days available to calculate each metric as further filtering is applied.The dotted lines in the top left plot display the estimates of $POD_{6,MED}$ using a season-wide fixed estimate of the parameters used to calculate $G_{s,O_3,MED}$. The column labeled "MED" displays results for all metrics calculated on days when $G_{s,O_3,MED}$ was available. The column labeled "PM" displays results for all metrics calculated on days when $G_{s,O_3,PM}$ was available.
